# The glucocorticoid receptor–autophagy axis in inflammation, immunoregulation, and cancer: a bidirectional regulatory network with therapeutic potential

**DOI:** 10.3389/fimmu.2026.1924727

**Published:** 2026-10-29

**Authors:** Xiuping Wang, Lingxiang Mao, Yujie Li, Jianjun Wang

**Affiliations:** 1 Department of Radioimmunology, Affiliated Kunshan Hospital of Jiangsu University, Kunshan, Jiangsu, China; 2 Jiangsu Key Laboratory of Medical Science and Laboratory Medicine, School of Medicine, Jiangsu University, Zhenjiang, Jiangsu, China; 3 Department of Clinical Laboratory, Affiliated Kunshan Hospital of Jiangsu University, Kunshan, Jiangsu, China

**Keywords:** autophagy, cancer, glucocorticoid receptor, immunoregulation, therapeutic targeting

## Abstract

Glucocorticoid receptor (GR) activation and autophagy are central regulators of cellular homeostasis and disease progression. However, the bidirectional interplay between GR signaling and autophagy remains incompletely understood, limiting context-specific insights and the translation of glucocorticoid-based therapies. This review synthesizes current knowledge of their molecular interactions, delineates the continuum from GR activation to the autophagy pathway, and discusses the integrated network governing their crosstalk. Notably, this axis exerts dual functions: it can promote survival or induce cell death, drive or resolve inflammation, and modulate therapeutic sensitivity depending on cellular context, disease state, and ligand characteristics. We review evidence on the GR–autophagy axis in immune cell function, cytokine dynamics, and inflammation control, and focus on its roles in inflammation and cancer pathogenesis, its therapeutic potential, and the challenges of clinical application. By integrating diverse research findings into a coherent framework, this review underscores the microenvironment’s influence on cancer regulatory mechanisms, highlights key therapeutic nodes, and advocates precise modulation of the GR–autophagy relationship as a promising therapeutic strategy.

## 1 Introduction

Glucocorticoids (GCs), including dexamethasone (DEX) and prednisone, are first-line anti-inflammatory and immunosuppressive agents. They are routinely prescribed for asthma and other inflammatory airway diseases, remain mainstay therapy in rheumatoid arthritis (RA) and other autoimmune conditions (1), are employed for transplant immunosuppression and graft-versus-host disease after stem-cell transplantation (2, 3), and are used in certain hematological malignancies (2). Their actions span inflammatory disease of the lung, gut, joints, and central nervous system (3). This breadth reflects their potency: GCs act primarily through the glucocorticoid receptor (GR), a highly conserved nuclear receptor regulating immune responses, metabolism, development, and stress adaptation (4–6). Nonetheless, significant adverse effects accompany clinical efficacy in inflammatory, autoimmune, and certain neoplastic conditions and, in some contexts, treatment resistance (4, 5). Response varies by tissue, cell state, receptor isoform, chromatin accessibility, DNA response elements, cofactors, and post-translational modification, rendering a simple transactivation/transrepression model inadequate (7). Resistance arises through altered GR isoforms or receptor downregulation, impaired chaperone and nuclear cofactor regulation, and MAPK-related signaling (8). These sources support metabolic adverse effects and resistance in general (9). Still, the evidence remains mechanistically convergent and insufficient to quantify clinical risk, leaving unresolved how GR translates a single ligand signal into the diverse outputs that determine why therapeutic efficacy and adverse outcomes diverge across patients.

Autophagy, an evolutionarily conserved lysosomal degradation pathway, governs cellular homeostasis, energy balance, and stress adaptation and substantially influences immune cell function, inflammation, and cell fate in physiological and pathological states (10–12). Initially characterized as a bulk degradation system for cytoplasmic components under nutrient deprivation, it is now recognized as a central regulator of innate and adaptive immunity, shaping antigen presentation, pathogen clearance, inflammatory signaling, and lymphocyte development and survival (10, 11). This immunological role provides the context in which the pathway intersects with GC signaling. Historically regarded as separate processes, lysosomal function and GR signaling have more recently been proposed to engage in reciprocal crosstalk governing homeostasis of the autophagy-lysosome pathway (ALP) (13, 14). The strongest direct evidence supports a lysosome-to-GR effect: chloroquine, bafilomycin A1, and TFEB knockdown inhibited lysosomal function or biogenesis, increased GR abundance, and enhanced GR-mediated repression of pro-inflammatory cytokines; chloroquine also enhanced GC treatment in a mouse arthritis model (10). GCs also act on autophagy in a dose- and duration-dependent manner, with low-dose GCs inducing osteocyte autophagy while high-dose or prolonged exposure promotes apoptosis (11). These findings establish lysosome-GR crosstalk and cell-specific GC effects on autophagic cell fate at the mechanistic and preclinical level.

The evidence remains incomplete. Cruz-Topete and Cidlowski (2015) (14) describe GR signaling as exerting dual anti- and pro-inflammatory effects, and Baschant and Tuckermann (2010) (12) review GR function in inflammation and immunity through conditional knockout and functionally selective models, but neither addresses autophagy. Kenney and Benarroch (2015) (13) overview the autophagy-lysosomal pathway without addressing GC-dependent regulation of it. None of these reviews quantifies clinical rates, and none establishes an integrated mechanistic model linking GC effects across immunity, bone, treatment resistance, and autophagy-lysosome regulation. Direct demonstration that autophagy drives clinical GC insensitivity or osteoporosis is likewise lacking, and studies on estrogen-autophagy crosstalk, cell-death pathways, inflammasome regulation, and disease-specific ALP activity offer relevant analogies rather than direct GR-autophagy evidence (15–17). Despite this evidence gap, its translational interest is nonetheless twofold: dysregulated autophagy may contribute to GC resistance and tissue damage, and the pathway is itself druggable. Resolving this gap requires a unifying mechanistic framework.

This review aims to integrate disparate findings into a coherent narrative and critically assess the current landscape. We first delineate the bidirectional regulatory network between the GR and autophagy, detailing the molecular mechanisms and dynamic interactions. Building on this foundation, we examine the axis’s role in immune regulation and cancer, and finally discuss the preclinical prospects and challenges of selective GR modulators and autophagy-targeted therapies.

## 2 The molecular continuum from GR activation to autophagy regulation

### 2.1 Overview of autophagy and its machinery

Autophagy is traditionally viewed as a catabolic degradation system involving lysosomal breakdown of cellular components within autophagosomes (18, 19). It is now recognized as a multifunctional signaling hub that contributes to diverse physiological and pathological processes (20). Whether autophagy is a direct effector of GR-related immunomodulatory function remains unresolved. The strongest evidence is directional rather than bidirectional: chloroquine, bafilomycin A1, or TFEB knockdown inhibited lysosomal function or biogenesis and increased GR abundance and GR-mediated repression of pro-inflammatory cytokines (10). The reverse direction, in which GR signaling governs autophagic flux, is proposed rather than demonstrated, and current work argues that transcriptional and cytoplasmic GR signaling may conditionally modulate the inflammatory response and intracellular homeostasis under stress (21).

Three major forms of autophagy have been characterized. Macroautophagy involves forming double-membraned autophagosomes and their subsequent degradation within autolysosomes (22). Microautophagy occurs via direct invagination of vesicular or endosomal membranes through mechanisms that can be endosomal sorting complex required for transport (ESCRT)-dependent or ESCRT-independent (23). Chaperone-mediated autophagy (CMA) selectively targets proteins containing a KFERQ motif for translocation through the lysosome-associated membrane protein 2A (LAMP2A)–HSPA8 complex into lysosomes (24, 25) (**Figure 1**). Although all forms contribute to cellular quality control, current understanding of GR–autophagy crosstalk primarily centers on macroautophagy. Consequently, in this article, we refer to macroautophagy simply as “autophagy”.

**Figure 1:**
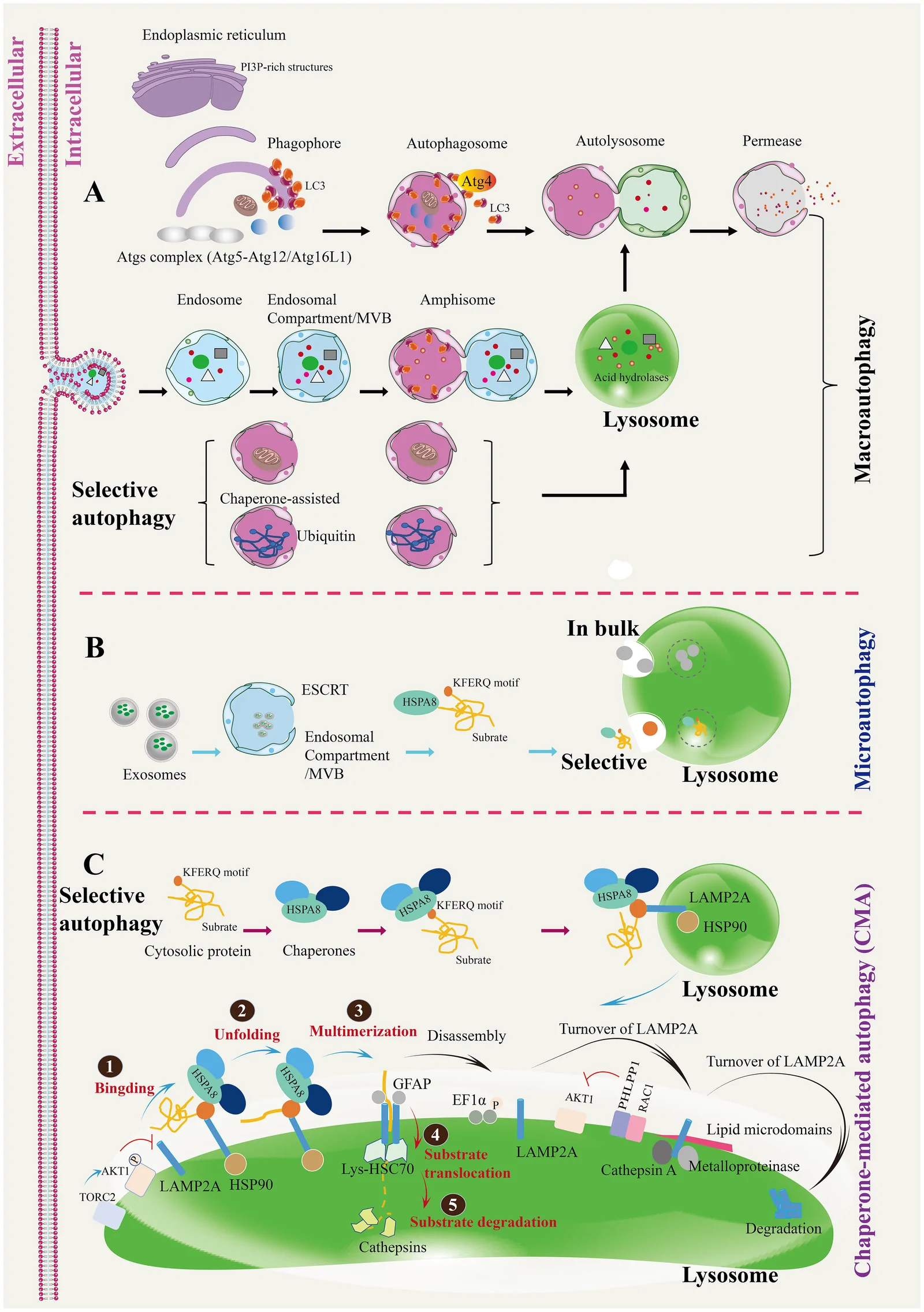
Overview of autophagy pathways. **(A)** Macroautophagy (MA): PI3P-enriched structures on the endoplasmic reticulum serve as initiation sites and the primary source of autophagosomes. Macroautophagy sequesters cytoplasmic cargo within a double-membrane isolation membrane via autophagy-related proteins (ATGs). Following LC3 recruitment to both membranes, the autophagosome closes and fuses with lysosomes to form autolysosomes, where cargo is degraded and recycled. Alternatively, the autophagosome may fuse with endosomes to form an amphisome before lysosomal fusion. Macroautophagy can be bulk or selective depending on the cargo. **(B)** Microautophagy (MI): Cytoplasmic components are directly invaginated into lysosomes. Selective microautophagy targets proteins via HSC70 in an HSPA8- and ESCRT-dependent manner for delivery to endosomal vesicles. **(C)** Chaperone-mediated autophagy (CMA): The chaperone HSPA8 guides KFERQ motif–bearing proteins to lysosomes, where LAMP-2A facilitates unfolding and translocation into the lumen. The process involves: 1) protein–chaperone complex binding to LAMP-2A; 2) substrate denaturation; 3) LAMP-2A multimerization to form a translocation complex; 4) substrate translocation mediated by lysosome-resident HSC70 (LYS-HSC70); and 5) completion of translocation, followed by LAMP-2A dissociation. TORC2 regulates LAMP-2A turnover via the Akt–PHLPP1 axis, along with membrane metalloproteinases and tissue proteinases in lipid microdomains. CMA activators and inhibitors are indicated in blue and red, respectively.

Autophagy proceeds through sequential stages: initiation (mediated by the ULK1 complex and the Beclin-1–PI3K Class III complex), elongation (involving ATG5–ATG12–ATG16L1 conjugation and LC3 lipidation), and maturation (regulated by Rab7 and SNARE proteins) (26). This multi-step machinery provides a versatile regulatory scaffold that extends beyond degradation, enabling nuanced integration with signaling pathways, including those driven by GR. Within this framework, the abundance of GR is linked to alterations in autophagy–lysosome biogenesis (**Figure 2**). Whether GR activation restores lysosomal function and rescues ALP dysfunction, thereby modulating hormonal sensitivity and inflammatory responses, is supported by a single mouse arthritis model (10) and by an argument that remains to be tested in human autoimmune disease (21).

**Figure 2:**
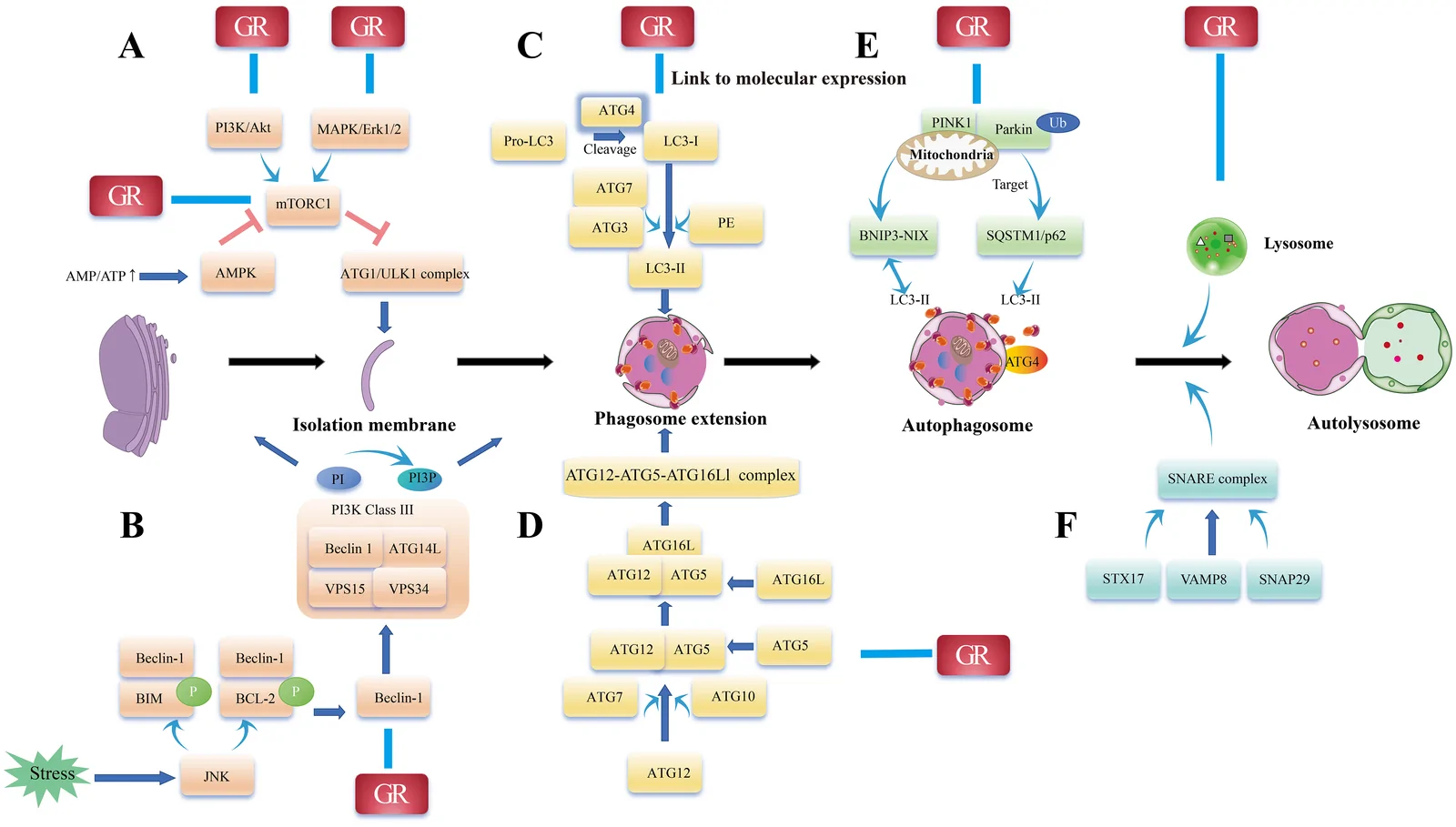
GR-implicated molecular pathways in autophagy regulation. The GR modulates key stages of autophagy (detailed in **Figure 4**). **(A)** Autophagy initiation: Upstream signals (PI3K/Akt, MAPK/ERK1/2) converge on the AMPK–mTORC1 axis. AMPK inhibits mTORC1, relieving ULK1 complex suppression and promoting autophagosome nucleation. **(B)** Beclin 1–VPS34 complex regulation: Stress-activated JNK phosphorylates BCL-2 and BIM, disrupting their inhibitory binding to Beclin 1. Released Beclin 1 activates the Class III PI3K complex (VPS34–VPS15–ATG14L) to generate PI3P for phagophore expansion. GR signaling can influence this step by modulating BCL-2 expression. **(C)** LC3 processing: Pro-LC3 is cleaved by ATG4 to form LC3-I, which is conjugated to phosphatidylethanolamine via ATG7 and ATG3 to generate membrane-associated LC3-II. **(D)** ATG5–ATG12–ATG16L1 conjugation: This E3-like complex is essential for phagophore elongation and LC3 lipidation. **(E)** Cargo recognition in selective autophagy: Receptors (BNIP3, NIX) and adaptors (SQSTM1/p62) target damaged organelles (e.g., mitochondria) for degradation. **(F)** Autophagosome–lysosome fusion: The SNARE complex (STX17–SNAP29–VAMP8) mediates fusion of autophagosomes with lysosomes, forming autolysosomes for cargo degradation.

### 2.2 GR structure and activation as the foundation for initiation signals

GR is encoded by the *NR3C1* gene on chromosome 5q31. *NR3C1* undergoes alternative splicing, yielding multiple isoforms; the two principal forms are GRα (~777 amino acids) and GRβ (~724 amino acids), with GRα acting as the canonical mediator of GC signaling (27). GRβ has a truncated ligand-binding domain and was traditionally viewed as an inhibitor of GRα-mediated signaling, but evidence now supports a more condition-dependent model in which GRβ regulates gene expression through both GRα-dependent and GRα-independent mechanisms, with independent transcriptional activity demonstrated *in vivo* (28, 29). In inflammatory settings, cytokines increase GRβ expression and the GRβ/GRα ratio, yet GRβ abundance is not established as a universal biomarker of steroid resistance (30, 31). We interpret this dissociation as evidence that the ratio is informative only in a disease- and tissue-specific manner, and that it cannot be used as a stand-alone index of GC responsiveness. In cancer, GRβ has been implicated in glioblastoma oncogenesis, whereas GC resistance in lymphoid malignancies remains multifactorial, so findings from one tumor type should not be extrapolated across cancers (32, 33). Studies of GR and autophagy report cell-type-dependent effects, but none isolates GRβ from GRα, so the contribution of GRβ to autophagic output remains undemonstrated (34, 35). Whether GRβ modifies GRα-dependent autophagic programs, and whether the GRα/GRβ balance predicts autophagic output independently of total GR abundance, remains an open and testable question; GRβ is therefore treated here as a candidate determinant of the direction of GR–autophagy crosstalk and revisited in Section 7. In the following text, “GR” refers to GRα.

Structurally, GR comprises an N-terminal transactivation domain (NTD/AF-1), a central DNA-binding domain (DBD) containing two zinc fingers, a hinge region, and a C-terminal ligand-binding domain (LBD) (36). These domains collectively determine receptor conformation, dimerization potential, and transcriptional competence (37–39) (**Figure 3A**). In the unliganded state, GR resides in the cytoplasm within multiprotein complexes that include chaperones such as heat shock protein 70 (Hsp70) and Hsp90, as well as co-chaperones like p23 and FKBP51/52 (40, 41). GC binding induces conformational rearrangements that promote nuclear translocation, GRE promoter recruitment, and transcriptional regulation of GCs response elements (GREs) (42). Beyond canonical genomic actions, ligand-bound GR also exerts rapid non-genomic effects at cytoplasmic and membrane sites, modulating signaling kinases and autophagy regulators independently of direct DNA binding.

**Figure 3:**
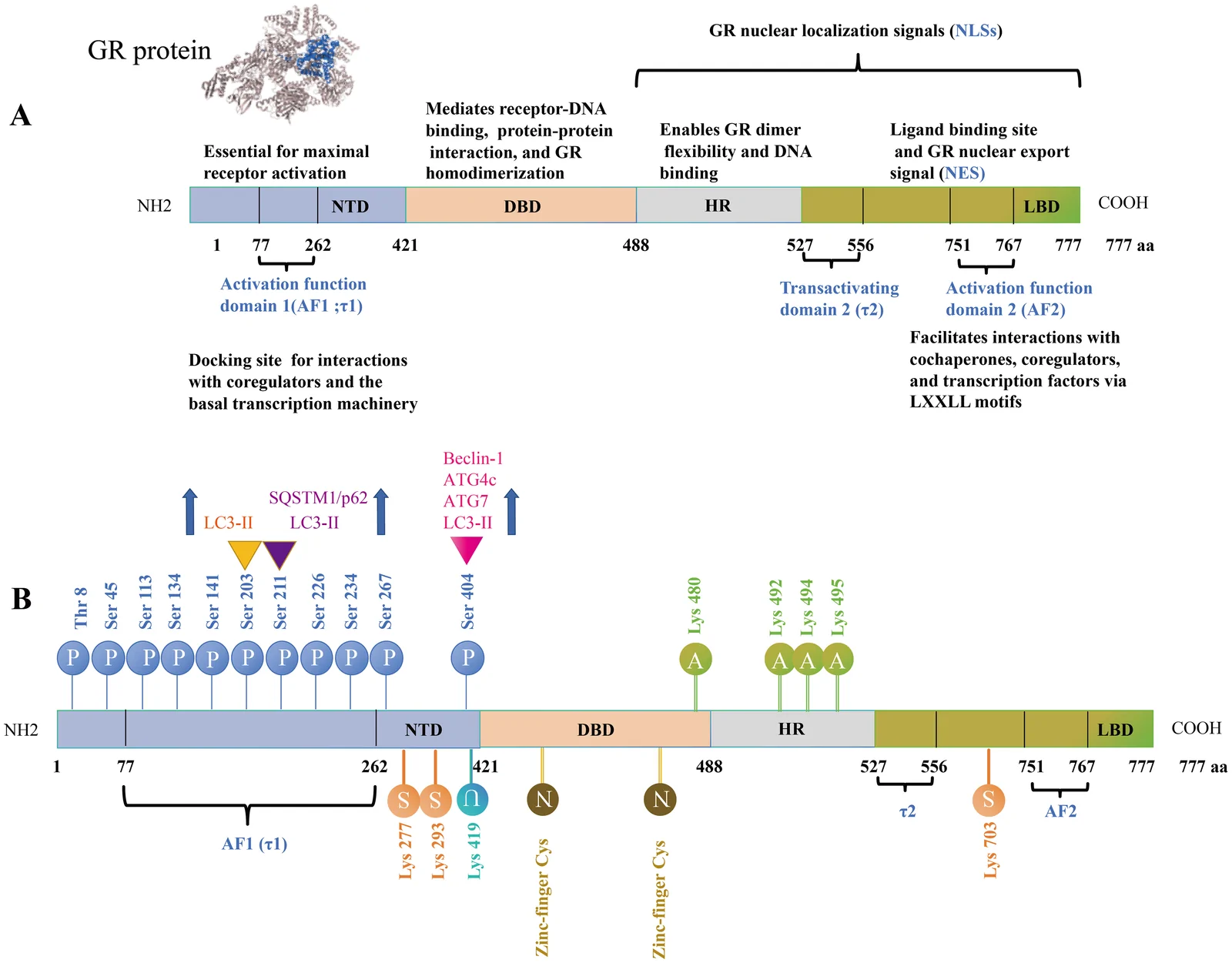
Structure of human GRα and its autophagy-related PTM sites. **(A)** Domain architecture of hGRα. The protein contains domains for receptor activation, dimerization, and chaperone interactions (e.g., heat shock proteins). It also harbors a nuclear localization signal (NLS), a nuclear export signal (NES), and an activation function (AF) domain regulating transcriptional activity and intracellular trafficking. **(B)** Autophagy-related PTMs of GRα. Phosphorylation at serine residues 203, 211, and 404 is linked to upregulation of autophagy-related proteins, including LC3-II, SQSTM1/p62, Beclin-1, ATG4a, and ATG7. GRα undergoes multiple PTMs—phosphorylation (P), sumoylation (S), ubiquitination (U), nitrosylation (N), and acetylation (A)—primarily on serine (Ser), threonine (Thr), and lysine (Lys) residues. These modifications modulate receptor stability, subcellular localization, and transcriptional activity.

In the nucleus, GR functions as a ligand-dependent transcription factor via two mechanisms (36, 43): (i) transactivation at positive GREs, often in cooperation with lineage-specific cofactors; and (ii) transrepression, either via binding to negative GREs or through protein-protein interactions with other transcription factors (TFs). The resultant transcriptional output is shaped by GR isoform expression, GRE sequence, chromatin accessibility, and the repertoire of co-regulators (44). Following GC stimulation, GR rapidly occupies pre-existing enhancer regions (44, 45), and subsequent enhancer remodeling is reported to regulate *ATG* expression and modulate autophagic flux, although whether this reflects preferential targeting of *ATGs* or a broader transcriptional response remains unclear (34, 46–50). Upon ligand withdrawal, GR returns to the cytoplasm, completing its cycle (51) (**Figure 4**). GR can also regulate gene expression indirectly by interacting with non-GR transcription factors, indicating regulatory pathways beyond direct GRE binding (52).

**Figure 4:**
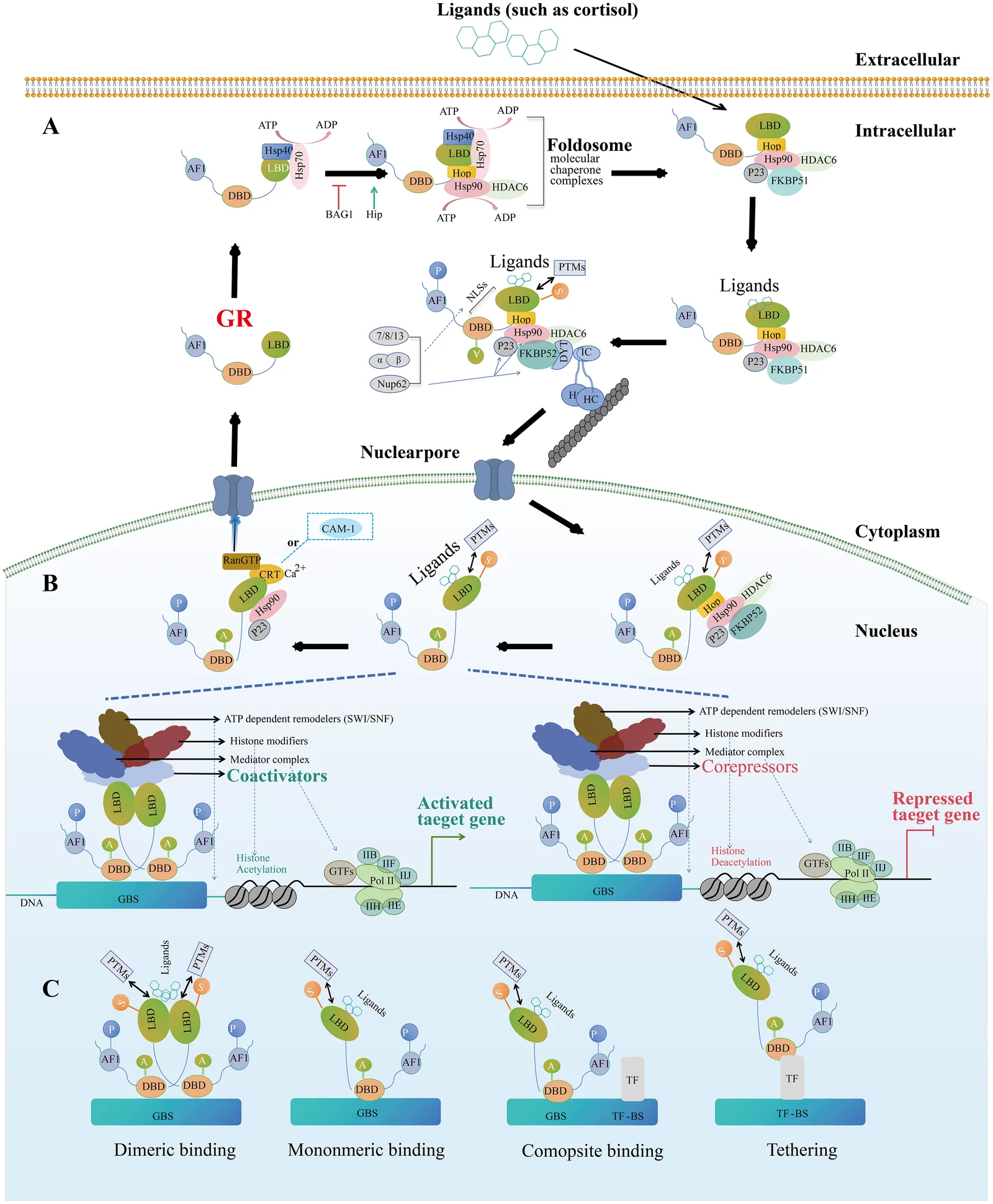
Cortisol/GC-mediated GR signaling pathway. **(A)** Receptor activation and chaperone-assisted maturation: GC binds unliganded GR, initiating a chaperone cycle involving Hsp70–Hsp40, Hop, Hsp90, p23, and FKBP51 to achieve a high-affinity GR conformation. FKBP52 subsequently exchanges with FKBP51, linking GR to the dynein–dynactin motor complex for nuclear trafficking. Importins and Nup62 mediate nuclear import via GR’s NLS; exportin 1 and calreticulin govern nuclear export **(B)** Nuclear events and transcriptional regulation: Ligand-bound GR bindsGCs response elements (GREs) and recruits: (i) ATP-dependent chromatin remodelers (e.g., SWI/SNF); (ii) histone modifiers that regulate methylation and acetylation of histones, GR, and associated factors; (iii) mediator and general transcription machinery (Pol II, TFIID, TBP); and (iv) co-regulators that fine-tune target gene expression. **(C)** Genomic binding and context-dependent regulation: GR binds as a dimer to classical GREs or engages half-sites with adjacent non-GR transcription factors (TFs). Cross-talk with non-GR TFs further modulates transcription. Post-translational modifications—phosphorylation (P), sumoylation (S), and acetylation (A)—modulate GR activity and DNA binding.

### 2.3 Core machinery translating GR signals into autophagy initiation

The autophagy initiation apparatus comprises Beclin-1-containing complexes, the ULK1 initiation complex, and the ATG conjugation systems, all orchestrated by nutrient- and energy-sensing pathways such as mTOR and AMPK (20, 53, 54) (**Figure 2**). GR integrates into and modulates these broader signaling networks, acting as a dynamic node that converges hormonal, metabolic, and stress signals to influence autophagy initiation (36, 44, 46, 55–57). The reported mechanisms differ substantially in evidential strength, ranging from transcriptionally defined axes supported by promoter-level and functional data to correlative associations confined to single experimental models (**Figure 5**; **Table 1**). We therefore classify them below according to the strength of evidence and the biological context in which they have been demonstrated.

**Figure 5:**
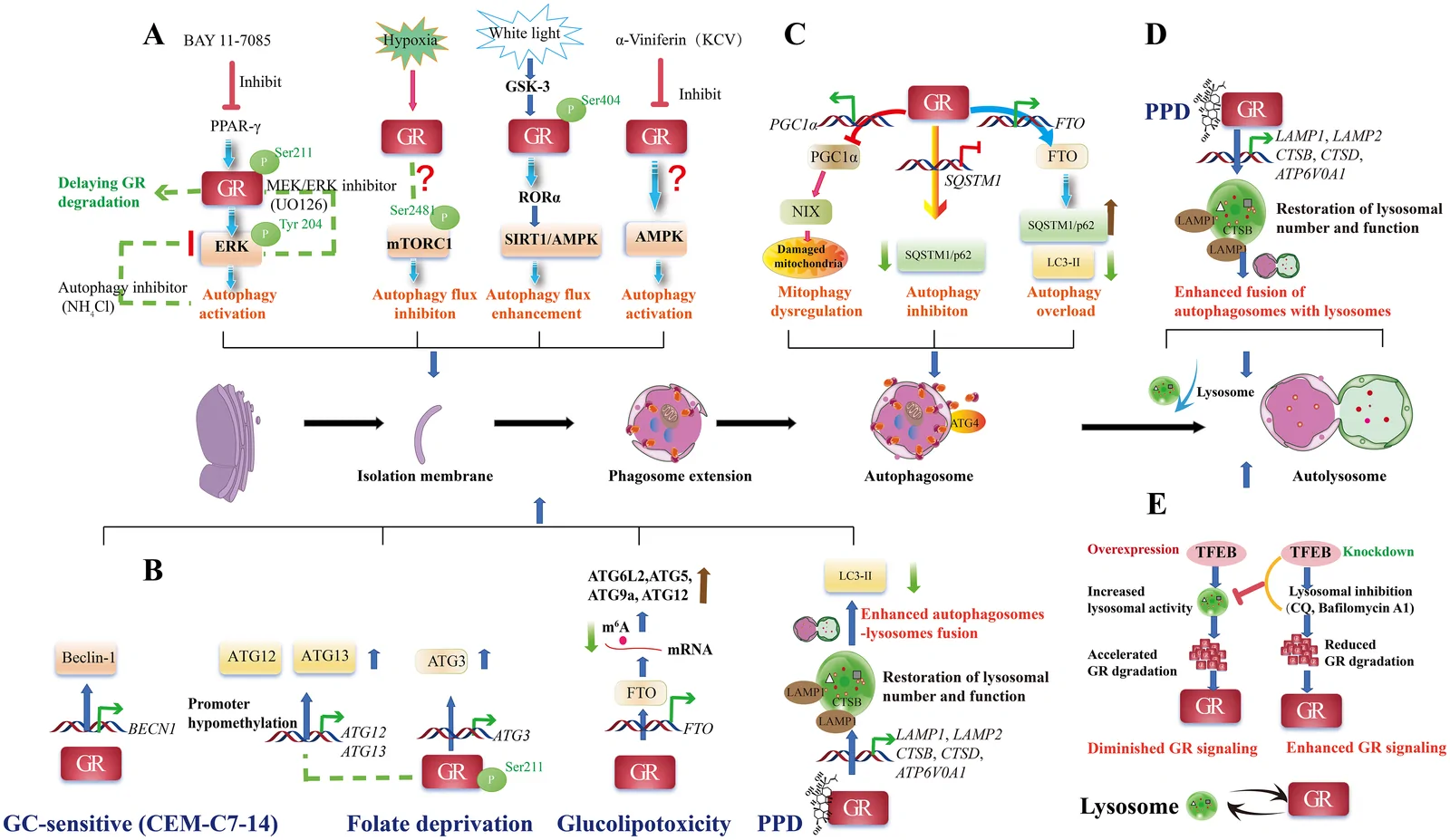
Effect of GR across autophagy stages. **(A)** Autophagy initiation: BAY 11-7085–mediated PPAR-γ inhibition induces GR Ser-211 phosphorylation and degradation. NH_4_Cl-induced autophagy inhibition reduces ERK phosphorylation, suggesting ERK mediates GR-regulated autophagy; UO126 (ERK inhibitor) suppresses autophagy and delays GR degradation, indicating a positive feedback loop. Under hypoxic conditions, GR sustains mTORC1 activity via phosphorylation at Ser-2481, thereby limiting autophagy. White-light LED irradiation promotes GR Ser-204 phosphorylation and nuclear translocation via GSK-3, thereby upregulating RORα and activating the SIRT1/AMPK axis. α-Viniferin suppresses GR and induces AMPK-dependent autophagy. **(B)** Autophagy elongation/processing: In GC-sensitive ALL cells, GR is recruited to the BECN1 promoter to regulate Beclin-1. Folate deficiency induces GR Ser-211 phosphorylation, promoting GR binding to the ATG3 promoter, with hypomethylation of ATG12 and ATG13. Under glucolipotoxicity, GR upregulates FTO, reducing m^6^A on ATG transcripts (ATG2, ATG12, ATG13, ATG9a) and stabilizing their mRNAs PPD-activated GR upregulates lysosomal genes (*LAMP1/2*, *CTSD*, *ATP6V0A1*), enhancing lysosomal degradation and reducing LC3-II/SQSTM1/p62 accumulation. **(C)** Selective autophagy: Stress-induced GR activation reduces PGC-1α expression, impairing NIX-dependent mitophagy. GR directly represses SQSTM1/p62 transcription. In glucolipotoxic conditions, GR decreases SQSTM1/p62, promoting hyperautophagy. **(D)** Lysosomal degradation: PPD-activated GR upregulates lysosomal gene expression (as in B), accelerating cargo degradation. **(E)** Lysosome–GR feedback: TFEB enhances lysosomal function and promotes GR degradation, attenuating GR signaling. TFEB knockdown or lysosomal inhibition (chloroquine, bafilomycin A1) stabilizes GR and potentiates its signaling, forming a bidirectional regulatory loop.

**Table 1:** GR-centered autophagy signaling networks classified by strength of evidence and biological context.

Pathway/ Relationship	Strength of evidence	Established mechanism and evidence system(s)	Context-dependent observations	Therapeutic implications	References
GR-driven BECN1/ATG transcription	Best supported; concordant across independent systems	Induces BIRC3 and BECN1/ATG transcription via GR promoter recruitment; shown in GC-sensitive ALL cells and neurons.	Marrow-mediated reversal of Beclin-1 suppression indicates dependence; human corroboration limited.	Informs GC-sensitivity stratification; autophagy status as a resistance determinant.	(58, 59)
GSK-3/GR/RORα axis	Established within one tissue class; unreproduced outside it	Induces autophagy via GSK-3→GR→RORα signaling; defined in hippocampal neurons.	Broader tissue relevance unclear; no human confirmation.	Potential neuroprotective target via stress-induced autophagy.	(56, 60)
GR-Ser211/ATG3 and epigenetic tuning	Established within one model; generality unproven	Enhances *ATG3* transcription via Ser211 phosphorylation and locus-specific hypomethylation; defined under folate deprivation.	Extension to other *ATG* genes or stimuli unresolved; not established in humans.	Hypothetical epigenetic modulation; requires validation.	(58)
GR-FTO-m^6^A axis	Documented but unproven generality; single context	GR upregulates FTO, enhancing *ATG* mRNA stability via m^6^A erasure; defined in glucolipotoxicity.	Dependence on GR per se versus GC exposure untested; no replication validation.	A strategy for epitranscriptomic modulation of autophagy; requires validation.	(34)
ERK/GR relationship	Documented but unproven causality; correlative	ERK activity correlates with GR phosphorylation and flux; evidence of feedback; defined in synovial fibroblasts.	Causality in GR for ERK-driven autophagy remains unproven.	Modulating this loop may adjust flux in stress- and inflammatory-related contexts.	(55, 56, 60).
FKBP4/GR/NRF2 axis	Mechanistically defined but model-restricted; generalization untested	FKBP4 sequesters GR, suppressing NRF2 and modulating flux; defined in cancer models.	Dissection derives from a single model; human evidence limited.	Disrupting FKBP4-GR or activating NRF2 could recalibrate autophagy.	(61)
GR-NRF2 redox crosstalk in infection	Proposed; indirect, model-specific	Pathogen-induced GR affects autophagy via NRF2; defined in *H. pylori* infection.	Relevance to other pathogens or diseases undetermined.	May alter host autophagy in specific infections.	(62)
SUMOylation/c-Myc/miRNA network	Upstream circuit well defined; downstream output inferred	SUMOylation stabilizes c-Myc to drive miRNAs that suppress GR and autophagy; defined in multiple myeloma.	Direct flux measures absent; functional role of ULK1 and contribution to DEX sensitivity unvalidated.	SUMOylation inhibition may restore GR signaling and promote autophagy, pending flux validation.	(63)
AMPK/mTORC1/ULK1 axis	Weakest; largely correlative, causality undemonstrated	May a metabolic regulator intersecting GR signaling.	No consistent direction across systems; GR effect on mTORC1 is pro-proteasomal under normoxia and pro-autophagic under hypoxia.	Combinatorial targeting may fine-tune flux in metabolic disorders.	(46, 55, 64–66)

Direct transcriptional regulation of autophagy genes by GR constitutes the best-supported tier. Early studies in hippocampal neurons show that GR enhances ATG expression and autophagy activation (56, 58). A defined axis operates through glycogen synthase kinase-3 (GSK-3), which phosphorylates GR at Ser404, driving upregulation of the circadian regulator RORα and subsequent activation of the pro-autophagic SIRT1/AMPK pathway, culminating in increased LC3-II, Beclin-1, ATG4c, and ATG7 levels (56, 60) (**Figures 3B**, **5A**; **Table 1**). Evidence for this GSK-3/GR/RORα axis includes GR phosphorylation-site mapping, promoter-level regulation of RORα, and downstream readouts of autophagic markers, although its validation has largely been limited to neuronal contexts. In neuronal models of folate deprivation, GR phosphorylation at Ser211 enhances transcriptional activity and promotes direct binding to the *ATG3* promoter, upregulating ATG3 expression, with concurrent increases in LC3-II and locus-specific DNA hypomethylation at GR-binding sites of other *ATG* genes (58) (**Figure 5B**). Disease-relevant models reinforce the physiological significance of GR-driven transcription: in GC-sensitive acute lymphoblastic leukemia (ALL) cells, DEX induces BIRC3 (cIAP2) expression and promotes GR recruitment to the *BECN1* promoter, events absent in resistant cells, and the bone marrow microenvironment can counteract chemotherapy-induced Beclin-1 suppression (59) (**Figure 5B**). The consistency of promoter occupancy, transcript induction, and autophagic readouts across independent neuronal and leukemic systems supports classification of GR-driven BECN1 and ATG transcription as well-established, while the GSK-3/GR/RORα and Ser211/ATG3 routes remain established within, but not yet beyond, their respective model systems.

A second tier comprises pathways for which mechanistic components are documented but whose generality is unproven. GR-dependent upregulation of the RNA demethylase FTO reduces m^6^A methylation on mRNAs encoding autophagy-related proteins such as ATG5 and ATG12 under glucolipotoxicity; This epitranscriptomic modulation enhances transcript stability and translational efficiency, leading to a dose-dependent increase in LC3-II and a decrease in SQSTM1/p62, thereby potentiating autophagic flux, as demonstrated using the mRFP-GFP-LC3 reporter and pharmacological verification of flux (34) (**Figure 5C**). This GR-FTO-m6A axis rests on a single disease context, and its dependence on GR per se, rather than on GCs exposure, has not been dissected genetically. Similarly, crosstalk with the ERK pathway is documented primarily in synovial fibroblasts, where the NF-κB inhibitor BAY 11–7085 induces autophagy before apoptosis, accompanied by GR phosphorylation at Ser211 and subsequent GR degradation; in this setting, ERK activation coincides with Ser211 phosphorylation and increased autophagic flux, whereas autophagy inhibition attenuates ERK phosphorylation, suggesting a potential feedback loop (55) (**Figure 5A**; **Table 1**). Notably, ERK-mediated GR regulation at these loci appears indirect, since GR transrepression depends on Ser246 phosphorylation by JNK rather than ERK, despite the capacity of ERK to phosphorylate GR *in vitro* (67); this dissociation concerns the repression function specifically and does not displace the Ser211 association with autophagic flux observed in fibroblasts (55) (**Figure 5A**; **Table 1**).

The weakest tier concerns the AMPK-mTORC1-ULK1 axis. Although AMPK activation and mTORC1 inhibition promote autophagy via ULK1 (64, 65), the links to GR are largely correlative, and no consistent directional relationship has been established across systems. In castration-resistant prostate cancer, GR downregulation correlates with AMPK activation and autophagy induction, implying that GR tonically inhibits this pathway (57). AMPK can phosphorylate GR at Ser211 (55), a modification associated with autophagy regulation in some contexts (55), but whether this phosphorylation is directly required for autophagic flux control remains to be validated. GR’s effect on mTORC1 activity also appears context-dependent: under normoxic conditions, GR signaling promotes 26S proteasome activation, whereas in hypoxia-induced muscle atrophy, GR associates with mTORC1 inhibition and facilitates autophagic proteolysis (46) (**Figure 5A**; **Table 1**). These observations identify GR as a modulator of the AMPK-mTORC1-ULK1 axis rather than a defined upstream regulator, and directional causality has yet to be demonstrated in any single system.

### 2.4 GR-mediated gene expression and GR post-translational modifications in autophagy regulation

GR influences autophagy via a transcriptional program that is modulated by GC signaling and by receptor post-translational modifications (PTMs). The best-supported mechanism is direct transcriptional control of core autophagy and mitochondrial-homeostasis genes by ligand-activated GR. In a neuronal model, stress-induced GC excess activates GR to repress PGC1α expression and its nuclear translocation, selectively suppressing BNIP3L/NIX-dependent mitochondrial autophagy and thereby promoting mitochondrial accumulation, synaptic dysfunction, and cell death (68). This represents direct transcriptional evidence for a GR–mitophagy axis. By contrast, GR occupancy at the SQSTM1/p62 promoter represses transcription only in specific cellular contexts (**Figure 5C**) (69), indicating that GR-dependent repression of SQSTM1/p62 is context-restricted rather than a general rule. In ischemic brain injury models, the pharmacological GR agonist 20(S)-protopanaxadiol (PPD) upregulates lysosomal and autophagy-related genes, including *Lamp1*, *Ctsd*, and *Atp6v0a1*, and enhances ALP activity (21). Because this evidence is pharmacological, it establishes GR-dependence of the response but not direct GR occupancy at these loci. Genetic evidence further supports a protective GR function: GR deficiency exacerbates autophagosome accumulation and lysosomal dysfunction, whereas GR agonists restore ALP function (**Figures 5B, D**). Together, these data support association and GR-dependence, but direct causality for most target genes has not been demonstrated.

Receptor PTMs, including phosphorylation, SUMOylation, and acetylation, shape the GR transcriptional program by altering GR stability, localization, and co-regulator interactions (47–50, 55, 70). These modifications may confer specificity on GR’s autophagic output, but the evidence remains largely correlative. In synovial fibroblasts, Ser211 phosphorylation precedes LC3-II accumulation across diverse autophagy-inducing conditions (55). This temporal association does not establish that Ser211 phosphorylation is required for autophagic flux, and its necessity remains untested by loss-of-function experiments. In *Helicobacter pylori* (*H. pylori*)-infected macrophages, infection induces a distinct GR phosphorylation pattern (increased pSer203, decreased pSer221) that accompanies nuclear accumulation and NRF2 interaction. GR knockdown reduces SQSTM1/p62 and LC3-II, whereas GR overexpression increases both, consistent with a pro-autophagic role (62) (**Figure 3B**). These gain- and loss-of-function data support a genetic contribution of GR to autophagic marker levels in this infection context, although whether GR acts causally on flux or on autophagosome number alone was not resolved.

Two interpretive problems require explicit attention. First, LC3-II accumulation is often equated with increased autophagic flux, which is not necessarily true. LC3-II reflects the steady-state number of autophagosomes and can increase through enhanced autophagosome formation, blocked autophagosome–lysosome fusion, or impaired lysosomal degradation (71, 72). Likewise, SQSTM1/p62 degradation is often used as a flux readout, but SQSTM1/p62 is itself transcriptionally regulated, so changes in SQSTM1/p62 levels can reflect altered synthesis rather than altered degradation. Accurate flux assessment therefore requires complementary assays, such as tandem LC3 reporters and lysosomal inhibitor-based flux measurements, rather than static marker quantification alone (72, 73). These methodological constraints are not merely technical, as they carry direct implications for interpreting the GR-autophagy literature: many studies cited in the preceding sections report LC3-II or SQSTM1/p62 changes without flux-resolving assays, and this limitation should be weighed when assessing the strength of the mechanistic evidence. In human material, the problem is compounded because autophagosome number in fixed tissue reflects a single time point and cannot directly report flux; accordingly, current recommendations emphasize the need for flux-based approaches in patient-derived samples before inferring autophagy status for stratification purposes (74). Second, the direction of GR effects on SQSTM1/p62 is apparently contradictory across models. Under some conditions, GR represses SQSTM1/p62 transcription (69), whereas GR knockdown reduces p62 and GR overexpression increases it in *H. pylori*-infected macrophages (62). Established GR–NRF2 crosstalk can resolve this contradiction through at least two distinct mechanisms. In one, DEX enhances GR recruitment to antioxidant response elements (AREs) without altering NRF2 chromatin binding, reducing CBP recruitment and histone acetylation at these sites and thereby attenuating NRF2-dependent transcription (75). In the other, GR interacts directly with ARE-bound NRF2 in a tethering configuration, repressing NRF2-mediated activation of individual target genes (76). Because SQSTM1 is an NRF2 target (77), the net p62 response depends on whether GR restrains or permits NRF2 activity at that locus, and on which of these mechanisms predominates in a given cell type. GR transcriptional direction itself flips with genomic and chromatin context and with the availability of additional factors (78, 79), and GR-dependent regulatory switching shapes downstream phenotypes in a condition-specific manner (80). The apparent conflict therefore reflects context-dependent switching between GR-mediated restraint and NRF2-driven SQSTM1 activation, rather than mutually exclusive mechanisms. Whether infection-derived co-signals, GR isoform composition, or GC exposure duration sets the switch remains undetermined.

Collectively, GR can influence autophagy initiation through transcriptional and post-translational mechanisms, but the supporting evidence derives from diverse systems (neuronal, fibroblast, leukemic, muscle, and cancer models) with little direct cross-validation. Locus-level causality is established only for the neuronal PPARGC1A–BNIP3L/NIX axis; elsewhere the data are predominantly pharmacological, genetic, or marker-based.

## 3 The dynamic crosstalk network between GR and autophagy

GR and autophagy engage in bidirectional crosstalk whose two directions differ in evidential strength. In the forward direction, GR integrates hormonal, metabolic, and stress signals to regulate autophagic flux through mechanisms ranging from transcriptionally defined axes to correlative associations (Sections 2 and 3.1; **Table 1**; **Figure 5**). In the reverse direction, autophagy controls GR turnover through lysosomal degradation, a smaller but mechanistically distinct body of evidence (**Table 1**; **Figure 5**).

### 3.1 Multilayer crosstalk signals regulating GR-mediated autophagy

Autophagy is governed both by the initiation network described above and by more distal circuits (**Table 1**; **Figure 5**). The immunophilin FKBP4 binds and sequesters GR, inhibiting nuclear translocation. This sequestration downregulates NRF2 and enhances autophagic flux, evidenced by decreased SQSTM1/p62 and increased LC3B puncta (61). However, this study did not establish the mechanistic link between reduced GR nuclear translocation and NRF2 protein downregulation, nor did it directly measure autophagic flux under FKBP4 or GR manipulation. Protein-interaction data combined with concordant autophagic readouts distinguish this axis from purely correlative associations, but the mechanistic dissection derives from a single experimental model, leaving its generalization untested. Crosstalk between GR and NRF2-mediated redox signaling may additionally modulate autophagic gene expression and flux, shaping cellular adaptation and contributing to therapy resistance in specific disease contexts (61, 62). This autophagy-context GR–NRF2 interaction operates at the level of redox signaling and autophagic gene expression, distinct from the transcriptional mechanisms by which GR restrains NRF2 activity at antioxidant response elements through reduced CBP recruitment and histone acetylation (75) or through direct tethering to ARE-bound NRF2 (76). Because this redox component rests on indirect, model-specific evidence, it is best regarded as a plausible extension of the FKBP4/GR/NRF2 axis rather than an independently established mechanism.

In multiple myeloma (MM), SUMOylation stabilizes c-Myc, which transcriptionally upregulates miRNAs; miR-130b suppresses GR expression, whereas miR-25 targets ULK1, together contributing to DEX sensitivity (63). Pharmacological SUMOylation inhibition reverses this network, enhances GR signaling, and may promote autophagy, as indicated by upregulation of ULK1, LC3B, and P27 (63). Here, GR does not directly regulate autophagy-related proteins; rather, GR expression and autophagy regulation are both governed by the upstream SUMOylation/c-Myc/miRNA axis. Direct measures of autophagic flux, such as the LC3-II/I ratio and p62/SQSTM1 degradation, are absent, so the functional role of ULK1 and its contribution to DEX sensitivity remain to be experimentally validated. This axis is therefore an upstream regulatory circuit with an inferred, rather than demonstrated, autophagic output.

### 3.2 Autophagy-mediated regulation of GC sensitivity via GR turnover and signaling

Autophagy acts as a dynamic rheostat for cellular GC sensitivity by controlling GR turnover. In synovial fibroblasts, induction of autophagic flux by serum starvation or prednisolone accelerates GR degradation, whereas blockade of autophagosome–lysosome fusion with NH4Cl or inhibition of upstream MEK/ERK signaling delays GR downregulation (55). This supports a negative feedback loop in which GR activation triggers autophagy that terminates the GR signal and thereby sets the intensity and duration of GC responsiveness.

The relationship is bidirectional. The autophagy–lysosome system regulates GR stability, since TFEB-driven lysosome biogenesis enhances GR degradation while lysosomal inhibition stabilizes the receptor. Conversely, GR supports the degradative machinery, and its absence impairs lysosomal function and autophagic flux (10, 21). A complete feedback loop therefore emerges, in which autophagy sets GC sensitivity by directing GR turnover while GR sustains autophagic capacity through transcriptional programs (**Figure 5E**).

Autophagy does not act unidirectionally on GC sensitivity but operates as a variable hub modulated by pathological, physiological, and environmental factors. In resistant lymphoid malignancies, pharmacological autophagy inhibition restores GC sensitivity (81). Crosstalk with MAPK signaling can either mitigate or exacerbate resistance depending on cellular context (82–84). In chronic inflammatory settings such as asthma, persistent GC exposure induces autophagy with consequences for disease progression (85), and environmental stressors such as ozone disrupt autophagy and epigenetic regulation to further shape GC responsiveness (85–87).

The core paradigm of autophagy-mediated GR turnover is well supported, yet its behavior across disease states remains unresolved. We propose that the dominant variable determining whether autophagy restores or reduces GC sensitivity is the duration of GC exposure relative to the autophagic capacity of the cell. Under this model, an intact autophagic-lysosomal system in the presence of short-term GC exposure favors GR degradation and signal termination, preserving GC sensitivity (55, 88). By contrast, sustained GC exposure in cells with compromised lysosomal function, as occurs in resistant lymphoid malignancies and chronic inflammatory conditions, leads to GR stabilization and accumulated autophagic stress, producing the resistance phenotype (21, 89). Within this framework, GR phosphorylation at S211/S226 (90, 91) and GRα/GRβ isoform composition (92, 93) act as secondary modifiers that modulate target gene selectivity through differential cofactor recruitment. CDK9-dependent phosphorylation of GRIP1 further illustrates how cofactor modification within the GR complex can dictate specific target gene activation (94). Because GR activation drives autophagy dysregulation in a cell-type-dependent manner (34, 95), this isoform- and phosphorylation-dependent selectivity may contribute to the divergent autophagic and inflammatory outputs of a single ligand across cell types, although direct evidence linking these modifiers to autophagy or inflammation remains limited.

## 4 The GR–autophagy axis in immune modulation and inflammatory responses

The GR–autophagy axis modulates cell differentiation, cytokine responses, and inflammatory resolution under physiological and therapeutic conditions (**Figure 6**).

**Figure 6:**
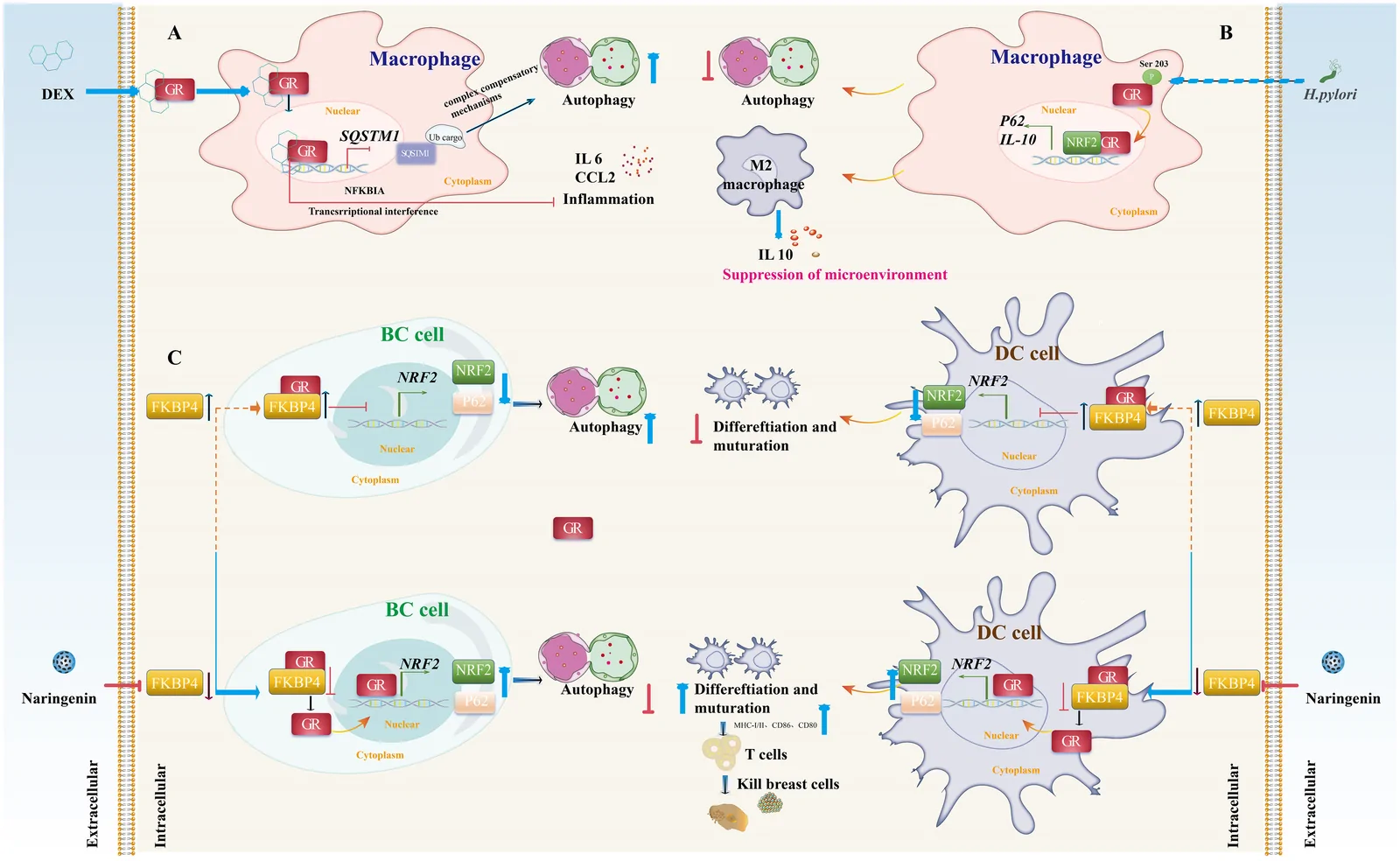
GR–autophagy axis in immune cell differentiation and cytokine secretion. **(A)** Immune-cell module (macrophages): GR-driven transcriptional control in macrophages. DEX suppresses *IL-6* and *IL-1β* transcription in a GR-dependent manner. Activated GR binds the SQSTM1 promoter to repress p62 expression; autophagy is nonetheless induced, suggesting compensatory regulatory mechanisms. **(B)** Microenvironmental module (macrophages in *H. pylori-*infected gastric cancer cell): *H. pylori*–mediated GR phosphorylation and immunometabolic remodeling. *H. pylori* infection induces GR Ser203 phosphorylation in macrophages, promoting GR–NRF2 interaction, upregulating p62 and IL-10, and associating with impaired autophagy and an immunosuppressive tumor microenvironment. **(C)** Tumor-cell-intrinsic versus immune-cell module (breast cancer): Naringenin modulation of GR–NRF2 signaling and immunity. Naringenin inhibits FKBP4, facilitating GR–NRF2 association, thereby suppressing autophagy and reducing breast cancer cell proliferation. Concurrently, Naringenin promotes dendritic cell differentiation and maturation, potentially enhancing antigen presentation and T cell–mediated anti-tumor immunity.

### 4.1 Immunomodulatory roles of GR-regulated autophagy in immune cell differentiation and function

GR-regulated autophagy is a key effector of GC-mediated immunomodulation, but its consequences diverge according to whether the autophagic response operates within tumor cells or within immune cells. In immune cells, GR-regulated autophagy shapes differentiation, macrophage polarization, and metabolic reprogramming; in tumor cells, the same axis governs cell-autonomous proliferation and survival and secondarily reprograms the local immune microenvironment.

GR influences autophagic flux, which in turn affects dendritic cell (DC) differentiation and function. Mechanistic studies show that altering autophagy biases DC maturation and antigen-presentation outcomes (12, 96, 97). The flavonoid naringenin modulates the FKBP4/GR/NRF2 axis to inhibit autophagic flux, an effect associated with promoted DC maturation (61); the tumor-cell arm of this same dataset is discussed below. This supports a causal link whereby GR-regulated autophagy shapes downstream immunophenotypes.

GR-mediated autophagy has been linked to macrophage functional reprogramming. In *H. pylori* infection, GR impairs autophagy and promotes M2 polarization and anti-inflammatory cytokine secretion in macrophages, while reducing apoptosis and elevating reactive oxygen species (ROS) production in gastric cancer cells, thereby creating a tolerogenic microenvironment and dampening pro-inflammatory signaling (62). The molecular mechanism by which the GR–autophagy axis governs M2 polarization nonetheless remains undefined.

The GR-autophagy link is also mechanistically defined in T lymphocytes. DEX promotes autophagy in GC-sensitive CD4/CD8^-^positive T cells by downregulating the Src kinase Fyn at both mRNA and protein levels, preventing Fyn-dependent phosphorylation of type I IP3 receptor (IP3R1) at Tyr353 and inhibiting IP3-mediated calcium signaling (98). In GC-treated lymphoid cells, autophagy is induced alongside apoptosis, and when apoptosis is blocked by Bcl-2 overexpression, autophagy prolongs lymphoma cell survival; 3-methyladenine inhibition of autophagy reduces this survival advantage, establishing autophagy as a Bcl-2-dependent pro-survival mechanism contributing to GC resistance (99). Autophagy in these cells is therefore not merely a downstream consequence of GC exposure but an active contributor to survival when apoptosis is blocked, which sharpens the unresolved question of whether autophagy protects or kills GC-treated lymphocytes. This divergence extends to metabolic reprogramming: GC-GR signaling promotes a shift toward fatty acid oxidation and mitochondrial respiration, metabolic states associated with regulatory and anti-inflammatory phenotypes, and this autophagy-mitochondrial remodeling contributes to the immunosuppressive microenvironment driven by GC action (10, 12, 68, 100, 101).

In tumor cells, by contrast, GR-regulated autophagy primarily affects cell-autonomous proliferation and viability rather than differentiation. Naringenin inhibits FKBP4, facilitating GR-NRF2 association, thereby suppressing autophagy and reducing breast cancer cell proliferation, while concurrently promoting DC differentiation and maturation and potentially enhancing antigen presentation and T cell-mediated anti-tumor immunity (61). Therefore, the same pharmacological input restrains tumor-cell growth by suppressing intrinsic autophagy and enhances immune-cell priming through DC maturation, indicating that tumor-intrinsic and immune-cell effects of autophagy manipulation can be mechanistically separable and directionally compatible. In *H. pylori*-infected gastric cancer cells, GR-mediated impairment of autophagy promotes M2 polarization and anti-inflammatory cytokine secretion (62); because this effect is exerted through the immune microenvironment rather than through tumor-cell-autonomous killing, this dataset illustrates a microenvironment-mediated rather than a cell-autonomous mechanism. Prevailing evidence further indicates that autophagy supports tumor-cell survival under metabolic and therapeutic stress (102, 103), consistent with predominantly cytoprotective rather than cytotoxic functions. Tumor-cell autophagy can additionally suppress immunogenic mechanisms associated with immunogenic cell death (ICD), including calreticulin exposure, dendritic cell maturation, and pro-T cell cytokine programs (104). These observations contrast with the double-edged nature of autophagy in shaping survival versus death programs and immune interactions (105, 106). Targeting autophagy is therefore anticipated to have compartment-specific net consequences, restraining tumor-cell fitness while potentially modulating immune-cell differentiation and effector mechanisms (102, 107, 108).

Taken together, the available evidence mechanistically defines the GR-autophagy axis in DCs, macrophages, and T lymphocytes, but the direction of its effect on lymphocyte fate is inconsistent. Direct evidence for GR-dependent autophagic regulation in regulatory T cells, CD8^+^ memory T cells, and B cells remains scarce, and whether the same apoptotic-threshold principle applies in these compartments is unresolved and warrants dedicated investigation.

### 4.2 Impact of GR-regulated autophagy on cytokine production, immune tolerance, and inflammation resolution

GR-regulated autophagy shapes cytokine secretion, immune tolerance, and inflammation resolution, and these processes together sustain immune homeostasis. A well-supported example involves the autophagy receptor SQSTM1/p62 and GR signaling. Mylka et al. showed that the selective GR modulator, compound A (CpdA), influences autophagy pathways, while DEX represses GR-dependent transcription of pro-inflammatory cytokines (e.g., IL-6 and IL-1β). Repression is reinforced by upregulation of anti-inflammatory genes and stabilization of NFKBIA/IκBα, indicating that selective GR ligands can enhance anti-inflammatory outcomes via complementary mechanisms whose relative contribution depends on the ligand and the cellular context (69). Pharmacological and genetic models support a functional requirement for autophagy in GC-mediated anti-inflammatory action. Autophagy inducers promote apoptotic clearance of macrophages and reduce pro-inflammatory cytokine production (109, 110). Conversely, *Atg5*- or *Atg7*-deficient mice show impaired GC-driven immune cell fate decisions and defective inflammatory control (111, 112). These loss-of-function data establish autophagy as necessary rather than merely correlative, although the specific autophagy-dependent step that governs GC-driven fate decisions remains to be mapped.

### 4.3 The GR–autophagy axis in immune suppression under stress and GC therapy

Under physiological stress, elevated endogenous GCs dampen excessive immune activation and protect tissues (113). The axis acts in this setting principally by suppressing pro-inflammatory cytokine production. Cortisol-induced autophagy in macrophages enhances containment of *Mycobacterium tuberculosis* (*Mtb*) by selectively reducing IL-1β and TNF-α, illustrating a context-specific mechanism to limit inflammation without indiscriminate immune suppression (114).

In therapy, GCs such as DEX stimulate autophagy and attenuate pathogenic inflammation. DEX-induced autophagy supports deletion or functional silencing of autoreactive T cells, contributing to GC efficacy in autoimmune and inflammatory diseases (98). In macrophages, GC-induced autophagy promotes degradation of pro-inflammatory mediators, mitigating cytokine storms. Canonical autophagy genes, including *ATG5* and *Beclin-1*, are frequently implicated (59, 112, 115). The net effect of this axis is not uniformly suppressive, and the variable that best explains the divergence is GC exposure duration. Acute or intermittent exposure engages autophagy as a cytoprotective, anti-inflammatory program: low-dose GCs induce autophagy in osteocytes and osteoblasts, with potential cytoprotective effects (11, 115, 116). Prolonged or high-dose exposure dysregulates the same pathway, which can impair host defense, promote immune exhaustion, compromise bone health, and in some lymphoid malignancies, contribute to treatment resistance, with autophagy inhibition restoring GC sensitivity (11, 81, 115, 116). Transcriptomic data indicate that GCs regulate stress-response programs, including autophagy-related genes such as *Eva1a* and *Plekhf1*, reflecting a coordinated response that integrates autophagy with broader cellular stress pathways (117). Upstream regulation via *SGK1*, *FKBP5*, and *NR3C1* supports tight control of immune activity and tissue protection, and GC exposure can modulate ROS, providing an additional regulatory layer linking stress signaling with autophagic activity and immune modulation (117). These findings identify candidate upstream regulators, but they remain correlative with respect to the GR–autophagy axis, and direct evidence that *SGK1*, *FKBP5*, or *NR3C1* causally set the autophagic threshold in immune cells is still lacking. Whether these regulators act upstream of autophagy or in parallel with it is therefore unresolved, and the distinction matters because it determines whether they can be targeted to shift the axis without disrupting canonical GR signaling.

Overall, GC actions engage multiple pathways, including hematopoietic cell apoptosis and cytokine suppression, with autophagy serving as a critical mediator of immune suppression (118).

### 4.4 Cross-regulation of GR-regulated autophagy in autoimmune and inflammatory responses

Within the immunosuppressive GC signaling network, GR-regulated autophagy provides a complementary mechanism that operates when canonical apoptotic pathways are compromised. In T cells, GC treatment can induce autophagy even when apoptosis is inhibited. Mechanistic data show that GCs increase LC3-II accumulation even with lysosomal inhibition, indicating autophagic flux, possibly via mTOR signaling suppression (118). Autophagy is therefore an integrated component of GC-mediated immunosuppression rather than a downstream bystander, and it may be engaged specifically when apoptosis is impaired.

The role of this axis in autoimmune and inflammatory disease is nonetheless unresolved. A prevailing model suggests that GR signaling uses autophagy to dampen inflammatory output and promote immune tolerance, but the net outcome depends on the disease model, tissue context, and the duration and intensity of GC exposure, and these dependencies have not been disentangled. We propose that the contradictions in this literature are best explained by a hierarchical model in which cell type sets the direction of the response, exposure duration sets its magnitude and reversibility, and disease state modifies the threshold at which a protective response becomes deleterious. Under this model, the divergent outcomes reported across autoimmune contexts are not evidence of a fundamental inconsistency in the axis itself but of comparisons made across different positions on this hierarchical axis. Therapeutic modulation of autophagy therefore retains potential for regulating immune responses in inflammatory disease (119), but its rational application requires disease-specific and duration-specific delineation of the GR–autophagy relationship rather than a single unifying assumption.

## 5 The GR–autophagy axis in inflammation and cancer: mechanistic insights and therapeutic opportunities

The GR–autophagy axis exerts regulatory functions in both inflammatory diseases and cancer, but the evidence base is predominantly preclinical and rests on experimental compounds (**Figures 7**, **8**; **Table 2**).

**Figure 7:**
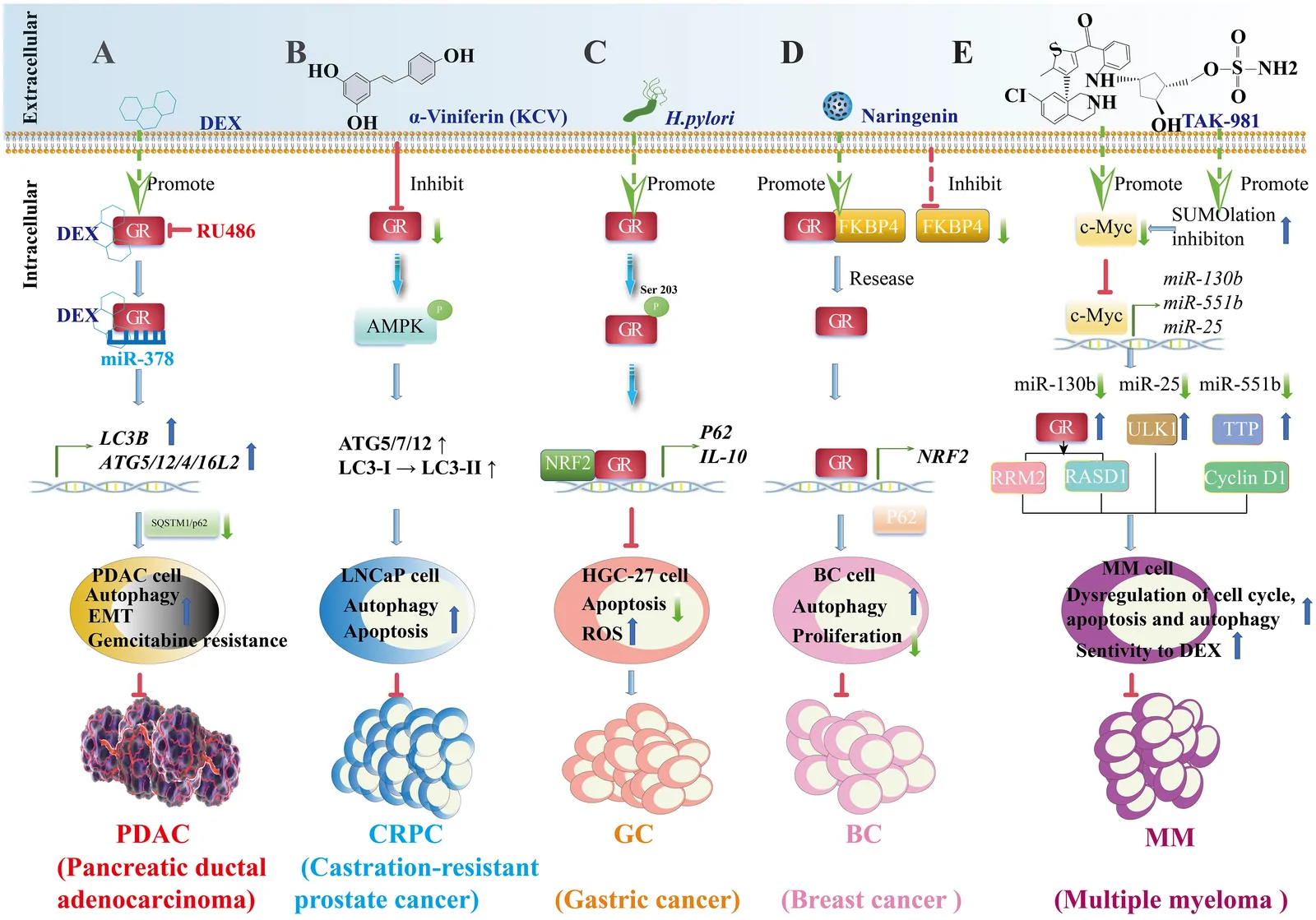
GR–autophagy axis in cancer. **(A)** In pancreatic ductal adenocarcinoma (PDAC), DEX-activated GR induces miR-378, upregulating ATG5, ATG12, and LC3B, enhancing LC3-I to LC3-II conversion and autophagic flux while degrading SQSTM1/p62, accompanied by EMT, stem-like traits, and gemcitabine resistance. RU486 mitigates these effects. **(B)** α-Viniferin (KCV) induces apoptosis in castration-resistant prostate cancer (CRPC) by inhibiting GR signaling and triggering AMPK-dependent upregulation of ATG5/7/12 and LC3 lipidation, leading to excessive autophagy and cell death. **(C)** In gastric cancer (GC), *H. pylori* increases GR expression and Ser203 phosphorylation in macrophages. Activated GR upregulates SQSTM1/p62 and IL-10, influencing GC cell apoptosis and ROS generation. **(D)** Naringenin downregulates FKBP4 in breast cancer (BC), promoting GR nuclear translocation. Nuclear GR stabilizes NRF2, increasing SQSTM1/p62, reducing autophagic flux, and suppressing BC proliferation. **(E)** In multiple myeloma (MM), the SUMO E1 inhibitor TAK-981 reduces SUMOylation and c-Myc, lowering miR-130b/551b/25. De-repressed GR promotes apoptosis via RRM2 and RASD1. Reduced miR-551b/25 elevates TTP, ULK1, and p27, perturbing cell cycle, apoptosis, and autophagy to enhance DEX sensitivity and suppress tumor growth.

**Figure 8:**
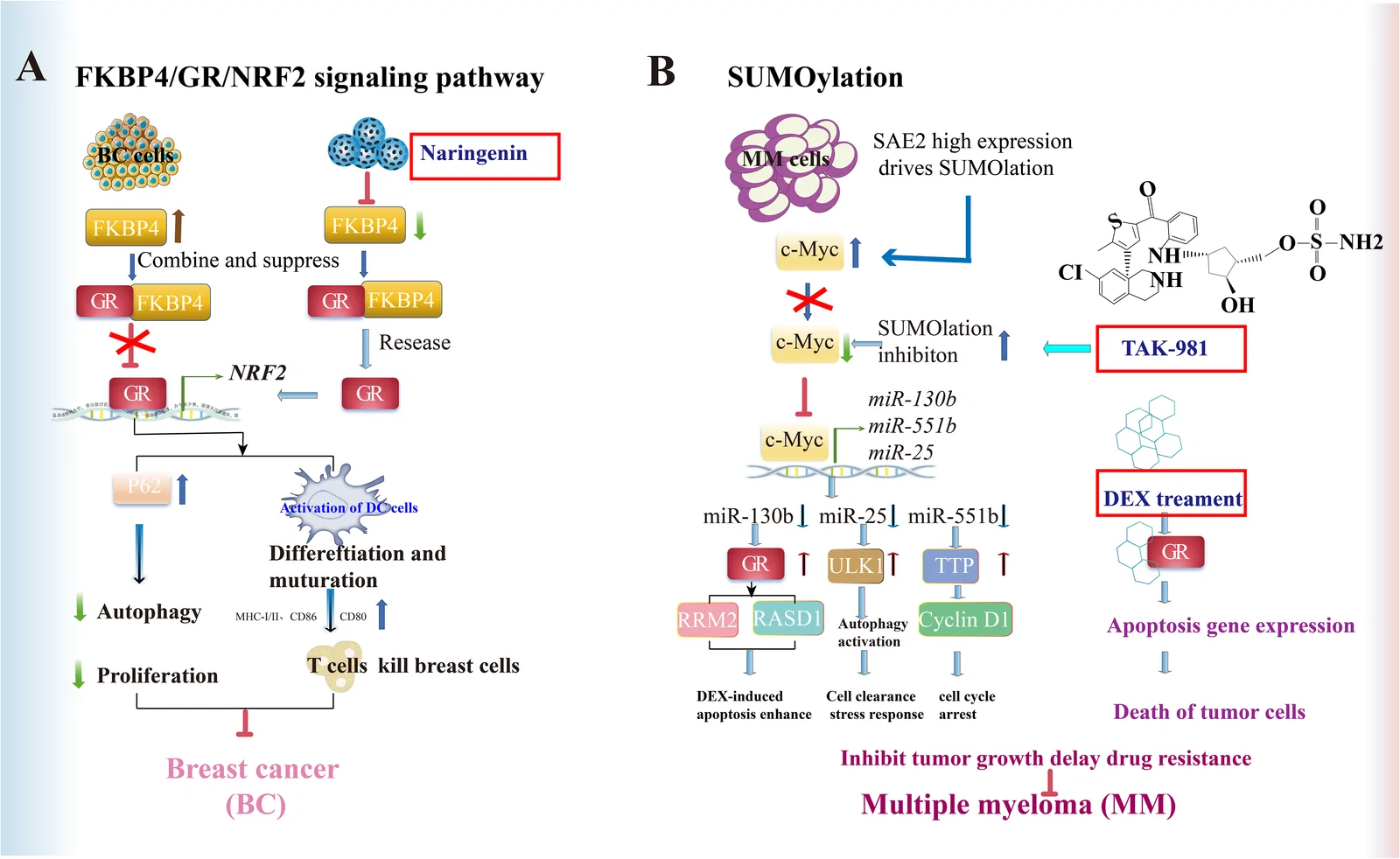
Therapeutic prospects of targeting the GR–autophagy axis in cancer. **(A)** Naringenin in breast cancer: High FKBP4 correlates with poor prognosis and suppresses GR and NRF2. Naringenin decreases FKBP4, relieving GR inhibition and activating NRF2 to inhibit autophagy and tumor growth. In the tumor microenvironment, Naringenin similarly reduces FKBP4, upregulates GR and NRF2, elevates dendritic cell maturation markers (CD11c, CD86), and promotes anti-tumor immunity. **(B)** SUMOylation blockade overcomes DEX resistance in multiple myeloma (MM): Elevated SAE2 enhances SUMOylation, activating c-Myc and miR-130b to suppress GR. TAK-981 inhibits SUMOylation, upregulating GR and ULK1 to promote apoptosis and autophagy, resensitizing MM cells to DEX.

**Table 2:** Therapeutic implications and potential interventions targeting the GR–autophagy axis.

Disease	Compound/ Intervention	Context-dependent observations	Potential strategies	References
Cancer	Autophagy inhibitors (e.g., CQ, HCQ)+GC; GR antagonists (e.g., RU486).	Resensitizes resistant cells in models; RU486 blocks pro-tumor autophagy in PDAC.	Overcome therapy resistance by inhibiting cytoprotective autophagy.	(81, 120–125)
	SUMOylation inhibitors (e.g., TAK-981).	Restores DEX sensitivity in myeloma models by reversing miRNA-mediated suppression.	Restore pro-death autophagy by targeting upstream modifiers.	(63)
	FKBP4 inhibitors (e.g., Naringenin).	FKBP4 inhibition activates GR/NRF2, suppressing tumor growth and promoting DC maturation.	Dual-action GR targeting minimizes systemic toxicity.	(61)
Autoimmune & inflammatory diseases	Selective GR modulators (e.g., CpdA); Natural compounds (e.g., solasodine).	Drive anti-inflammatory outcomes via autophagy-related pathways in models.	Modulate autophagic flux for anti-inflammatory resolution.	(69, 126–128)

### 5.1 The GR–autophagy axis in autoimmune and inflammatory diseases: balancing immune homeostasis and pathology

GC signaling through the GR exerts its immunosuppressive effects in part by modulating autophagic flux, which regulates inflammasome activity and pro-inflammatory cytokine expression in macrophages and dendritic cells (69, 118). In classical autoimmune diseases, including multiple sclerosis (MS), RA, and systemic lupus erythematosus (SLE), autophagy supports core immunological processes, including antigen presentation, lymphocyte survival, and cytokine balance (22, 119). Dysregulated autophagic flux has been mechanistically linked to aberrant interferon signaling and autoantigen modification (22), and GR signaling interacts with autophagy to modulate immune cell apoptosis and inflammatory output (96, 129). The disease specificity of these observations varies, limiting the claim that the axis constitutes a therapeutic node. In MS, RA, and SLE, the evidence is largely correlative, based on expression data and autophagy gene polymorphisms associated with disease susceptibility rather than interventional studies in patients (22, 119).

Tissue-specific inflammatory responses reveal a more consequential problem: the axis’s direction depends on the disease and, in at least one setting, is therapeutically unfavorable. In osteoarthritis (OA), reduced autophagic flux correlates with disease progression, and experimental models indicate that GCs can induce enduring changes in cartilage homeostasis: DEX impairs autophagic flux in fetal chondrocytes by epigenetically upregulating miR-1912-3p and downregulating the lysosomal enzyme CTSD (130). This finding is directionally opposite to the anti-inflammatory premise of Section 4.3, since here a GC suppresses rather than engages protective autophagy in a target tissue. We interpret this as evidence that the disease state, rather than cell type or exposure duration alone, determines whether GR engagement is protective, and the OA data derive from fetal chondrocytes and therefore require confirmation in adult cartilage before the mechanism can be assigned clinical weight. In allergic asthma, by contrast, GR-dependent anti-inflammatory effects of compounds such as solasodine involve modulation of autophagy-related signaling through FOXO3A, consistent with a protective role in airway inflammation (126).

Therapeutic implications are limited by the pharmacological properties of the available agents. Conventional GCs are potent but non-specific, and their off-target effects constrain long-term use. Selective GR modulators such as Compound A offer a strategy to engage beneficial GR–autophagy interactions with reduced off-target activity, but this evidence derives from cell-based and preclinical systems (69). No selective GR modulator has yet demonstrated efficacy in autoimmune disease through an autophagy-dependent mechanism in patients, and none has been shown to produce a validated change in an autophagy biomarker. Combining targeted GR modulation with autophagy-focused interventions for MS, RA, or SLE is therefore a rational hypothesis rather than a supported clinical strategy, and it requires human biomarker validation and phase I toxicity data before it can be advanced (22, 69, 118).

### 5.2 The GR–autophagy axis in cancer: from cancer pathogenesis to therapeutic intervention

#### 5.2.1 The GR–autophagy axis in cancer pathogenesis

In cancer, the GR–autophagy axis can promote tumor cell survival and therapy resistance by enhancing autophagic flux, or, under certain conditions, drive autophagic cell death and tumor suppression (120–123) (**Figure 7**). The variable that best explains these opposing outcomes is the metabolic and therapeutic context in which GR acts, rather than the tumor type itself. Where GR engagement supports existing cytoprotective autophagy, the axis promotes resistance; where GR suppression releases an unrestrained autophagic response that exceeds the cell’s degradative capacity, the outcome is cytotoxic. We use this distinction to organize the evidence below.

In many advanced cancers, GR signaling is co-opted to sustain autophagy, enabling adaptation to hypoxia, nutrient scarcity, and chemotherapy. In pancreatic ductal adenocarcinoma (PDAC), GCs activate GR and upregulate miR-378, which enhances core autophagy genes such as *ATG5*, *ATG12*, and *LC3B*, promoting LC3-II accumulation, SQSTM1/p62 degradation, and contributing to gemcitabine resistance. Pharmacological GR inhibition with RU486 reverses these pro-autophagic and chemoresistant features (122, 123). These findings establish a coherent preclinical mechanism, but they derive from cell lines and xenograft models, and RU486 is a progesterone and GR antagonist whose endocrine toxicity makes it unsuitable for chronic use in this indication. Whether GR inhibition can be achieved in patients at doses that reverse autophagy-dependent resistance without unacceptable endocrine effects remains untested. The opposing outcome is illustrated in castration-resistant prostate cancer (CRPC), where α-viniferin (KCV) downregulates GR, activates AMPK, and increases ATG5, ATG7, ATG12, and LC3-II. Although mTOR activity rises modestly, autophagy in this setting is predominantly AMPK-driven and is associated with apoptotic death, indicating a cytotoxic rather than cytoprotective autophagic outcome (57). The mechanistic contrast with PDAC is instructive: in PDAC, the axis reinforces an already protective autophagic program, whereas in CRPC, GR loss removes a constraint and permits an autophagic response that terminates in cell death.

Beyond tumor cell–intrinsic effects, the GR–autophagy axis shapes the tumor microenvironment. In *H. pylori*–associated gastric cancer, macrophage GR upregulation can foster an immunosuppressive milieu (62). In breast cancer models, the FKBP4/GR/NRF2 axis modulates autophagic flux, potentially affecting tumor–stroma interactions (61). Endothelial GR deficiency increases autophagic flux and activates pro-angiogenic Wnt/β-catenin signaling, promoting tumor progression (131, 132). At the molecular level, GR interfaces with the core autophagy machinery (LC3/ATG), energy-sensing pathways (mTOR, AMPK), and the Wnt/β-catenin pathway, producing tissue-specific outcomes (34, 123, 132, 133). PTMs, notably SUMOylation, add a further regulatory layer. In multiple myeloma, SUMOylation stabilizes networks that suppress GR expression and autophagy, contributing to DEX resistance; inhibiting SUMOylation can restore sensitivity by lowering miRNAs (e.g., miR-130b) that repress GR and autophagy genes (63, 134). These SUMOylation findings derive from cell-based models and identify a candidate mechanism rather than a validated target, and no inhibitor of this pathway has been tested in patients for the purpose of restoring GC sensitivity through autophagy.

#### 5.2.2 Therapeutic targeting of the GR–autophagy axis in cancer

Therapeutic strategies targeting the GR–autophagy axis remain largely preclinical and aim primarily to counteract cytoprotective autophagy that contributes to therapy resistance. In these contexts, combining GCs or selective GR modulators (SGRMs) with pharmacological autophagy inhibitors such as chloroquine derivatives has shown promise, including in lymphoma models in which autophagy-mediated survival pathways are disrupted (81, 127). A mechanistic basis exists for hematological malignancies: inhibition of autophagy can upregulate GR expression and restore GC sensitivity, potentially via modulation of the PI3K/AKT/mTOR axis (135). However, these observations derive predominantly from *in vitro* systems, and whether the same relationships hold in patients remains untested.

In tumors where autophagy exerts a tumor-suppressive function, strategies that preserve or enhance autophagic flux may be advantageous (136). This underscores the need for precise, tumor-specific modulation of the axis, since the direction of the therapeutic effect is dictated by the underlying autophagic role rather than by the pathway itself. Upstream regulation provides additional, though still experimental, points of intervention. SGRMs enable fine-tuning of GR transcriptional activity to steer autophagic responses in preclinical models (127, 128). Inhibiting specific regulatory nodes, such as SUMOylation (e.g., with TAK-981), concurrently alleviates GR suppression and pro-death autophagy in multiple myeloma (63, 128). A further example is breast cancer, where targeting the upstream co-chaperone FKBP4 (e.g., with Naringenin) promotes GR nuclear translocation; through the NRF2 pathway, this activation can suppress tumor-promoting autophagy and proliferation while enhancing dendritic cell maturation in the tumor microenvironment (**Figure 8**). The pharmacological specificity of these compounds is incompletely characterized, and their selectivity for GR- versus non-GR-mediated effects has not been systematically established.

A major clinical challenge is acquired GC resistance driven by GR downregulation, particularly in relapsed ALL (137–139). In these settings, strategies to restore GR function or exploit synthetic lethality are critical. The synergistic combination of autophagy inhibitors with GCs, which can mitigate the consequences of GR loss, provides a rationale for treating these malignancies, although this combination has not yet been evaluated in controlled clinical trials (135, 139–141).

Translation of these concepts into clinical practice will require robust biomarkers that are presently unavailable. Patient stratification needs to integrate GR status, functional autophagic flux, and molecular determinants (e.g., signaling pathway activity, miRNA profiles) to predict whether autophagy is pro-survival or tumor-suppressive within a given tumor (127, 136). None of these candidate biomarkers has yet been prospectively validated, and functional measurement of autophagic flux in patient samples remains technically challenging (74). Future trials should enroll biomarker-enriched cohorts to ensure precise targeting and minimize unintended effects (**Table 2**); such trials have yet to be designed or reported.

Across diverse pathologies, from cancer to autoimmunity, the GR–autophagy axis emerges as a regulator of cellular fate whose dysregulation represents a common pathogenic thread. Although preclinical evidence supports the concept of calibrated, tissue-specific modulation, clinical validation of efficacy, biomarker-based patient selection, and safety remains outstanding. Notably, systemic modulation of autophagy carries substantial risk of toxicity, given the homeostatic roles of autophagy in normal tissues, and long-term safety data for autophagy inhibitors in combination regimens are limited (142). Achieving the therapeutic promise of this axis will therefore depend on demonstrating target engagement, selectivity, and tolerability in adequately powered human studies rather than on extrapolation from model systems.

## 6 The challenges and prospects of the GR–autophagy axis in clinical applications

The GR–autophagy axis offers broad translational potential across oncology, autoimmune, and inflammatory diseases. However, its clinical application demands careful navigation of effects, dynamic autophagic flux, and patient heterogeneity. Despite opportunities to leverage GR–autophagy crosstalk to shape therapeutic responses, several formidable challenges constrain translation into routine care (142, 143).

### 6.1 Key determinants of the direction of GR–autophagy crosstalk

The net outcome of GR–autophagy crosstalk cannot be reduced to a single directional rule. Cell type determines which autophagic machinery is expressed and which GR transcriptional programs predominate, as immune cells, hepatocytes, osteocytes, and tumor cells differ in basal autophagic flux and lysosomal dependence. Ligand identity further shapes the response, since DEX and selective GR modulators (SEGRMs) differentially engage transrepression and transactivation (144). In macrophages, DEX and compound A both induce autophagy but with distinct characteristics, and the anti-inflammatory action of compound A requires the autophagy receptor SQSTM1/p62 (69). These findings support ligand-dependent differences, yet the transrepression/transactivation balance remains too simple to define autophagic direction (144).

Dose and exposure duration are the best supported modulators. In osteocytes, GCs induce protective autophagy at lower doses, whereas higher doses or prolonged exposure promote apoptosis (11, 145, 146); tumor necrosis factor α in the same model induced apoptosis but not autophagy (145). GR isoform composition remains plausible but unconfirmed: GRα binds GCs, and GRβ lacks a complete ligand-binding domain and may oppose GRα, while cytokines raise the GRβ/GRα ratio, although GRβ physiology is debated (30). Whether this ratio determines autophagic output is not established.

Metabolic signaling may add a further layer. In retinal pigment epithelial cells, GC exposure altered AMPK/mTOR signaling and increased autophagy, and GR antagonism abolished these effects (95). A liver study linked PI3K, lysosomal degradation, and GR function during fasting, without establishing a role in autophagic direction (89). Thus, cell type, dose and duration, ligand properties, isoform composition, and metabolic state are candidate modulators requiring validation in each experimental context, with dose and duration in osteocytes (11, 145, 146) and ligand properties in macrophages (69) the most directly supported. However, no retrieved study directly linked GR isoforms to reversing autophagic direction (30, 69).

### 6.2 Key challenges

Cell- and pathway-specificity. GC regulation of autophagy is not uniform. In a T-lymphocyte model, DEX promoted autophagy by downregulating Fyn and suppressing IP3-dependent calcium signaling, whereas a separate lymphocyte study found that BCL-2 inhibited GC-induced apoptosis without blocking GC-induced autophagy (98, 147). Responses therefore vary by cell type and signaling state, and the relationship between autophagy and apoptosis complicates systemic autophagy modulation.

Risks of prolonged GC exposure. Long-term GC use is associated with adverse metabolic effects, and GC-dependent transcriptional responses vary across individuals and cell types (148). Reducing systemic exposure and improving tissue selectivity remain key development goals.

Limited validation of target-engagement biomarkers. LC3 conversion and SQSTM1/p62 degradation have been used to characterize GC-associated autophagy in cell studies, but their ability to measure autophagic flux in patients or predict long-term outcomes is unestablished (81). Pharmacogenomic and biomarker-guided GC treatment is likewise investigational, with no broadly accepted, clinically actionable genetic markers (149).

Inter-individual variability. Studies using cells from multiple individuals have identified individual- and cell-type-specific GC-dependent transcriptional and GR-binding profiles, some associated with variants affecting GR or cofactor binding (148). This variability warrants consideration in GR–autophagy studies, although current evidence does not support treating autophagy-gene polymorphisms as established clinical predictors.

### 6.3 Strategic directions

Develop tissue- or cell-type-selective interventions. Candidate therapies should be evaluated across relevant cellular contexts, with attention to how GCs, GR modulators, and autophagy interventions interact, after which targeted delivery or local administration could be assessed for improved therapeutic window. In macrophages, the CpdA and DEX differentially regulated SQSTM1/p62 and autophagy, supporting the inclusion of cell context in candidate evaluation (69).

Build combination strategies around testable mechanisms. In GC-resistant lymphoid malignancy models, chloroquine or 3-methyladenine increased DEX-induced cell death, and Beclin-1 knockdown increased GC sensitivity *in vivo* (81). In childhood ALL, autophagy-dependent necroptosis was associated with reversal of GC resistance, and rapamycin plus DEX triggered this cell death pathway (150). These preclinical findings support testing combinations according to tumor type, resistance mechanism, and autophagy function rather than assuming clinical efficacy.

Treat biomarker candidates as exploratory. LC3-II/LC3-I, SQSTM1/p62, and GR-responsive readouts such as FKBP5, GILZ, and PER1 may serve as candidate measurements, yet the retrieved literature validates neither a composite GR–autophagy engagement index nor the use of circulating monocytes or tumor tissue as reliable proxies for *in vivo* autophagic flux. Assay performance, sampling time, correspondence with functional flux, and association with clinical outcomes require evaluation in a defined disease context; precision GC treatment remains an evolving research area (149).

Directly test proposed combinations before clinical translation. Evidence supports autophagy inhibition in some preclinical models of GC-resistant lymphoid malignancy, but it does not establish the efficacy of a SEGRM combined with a lysosomal inhibitor in GC-resistant multiple myeloma (81). This combination is therefore a testable hypothesis. Initial studies should assess activity, dependence on GR and autophagy-related mechanisms, and therapeutic window in multiple myeloma cell systems, patient-derived models, and appropriate *in vivo* models before clinical evaluation.

## 7 Discussion

GR signaling and autophagy form a coupled axis in immune responses and disease. GR modulates autophagy through transcriptional control, signaling crosstalk, and feedback, whereas autophagy governs GR stability and activity. These interactions underpin immune cell function and are implicated across cancer, inflammatory diseases, and autoimmune disorders. Translation remains difficult because crosstalk direction depends on measurable variables rather than a single mechanism, and current models rarely reproduce the cell types and disease states relevant to clinical decisions. Several questions remain unresolved. Whether GRα and GRβ differ functionally in autophagy regulation remains untested, as no study has compared the two isoforms within the same system; isoform-selective knockdown or knock-in models coupled with flux-resolved readouts would determine whether the GRα/GRβ ratio, rather than total GR, predicts autophagic direction under GC exposure. Whether the FKBP4/GR/NRF2 and SUMOylation/c-Myc/miRNA axes converge at a shared node is likewise unknown, although both act on NRF2 and c-Myc; mapping them onto one network would indicate whether NRF2 or c-Myc is the more tractable target. Whether sex- and age-related differences in GC pharmacokinetics and autophagic capacity shift the threshold at which GR signaling activates or suppresses autophagy requires stratification by sex and age with autophagic flux as an endpoint, particularly given the age-related decline in lysosomal function. Whether single-cell and spatial approaches can resolve the cell-type heterogeneity that recurs throughout this literature also remains open, since bulk analyses average opposing responses and leave the populations driving autophagic responses, and separating benefit from adverse effects, unidentified. Three methodological and translational priorities follow. Bidirectionality should be quantified through flux-resolved measurements in defined cell populations rather than static markers. Therapeutic development should favor agents that exploit the variables governing the axis, such as SEGRMs and lysosomal modulators, over broad autophagy inhibitors. Biomarker discovery should be embedded prospectively within GC-based trials, so that composite readouts of autophagic flux and GR target signatures can be validated against clinical outcomes. Addressing these questions would benefit predicting and selectively modifying GR-autophagy responses in patients.

## References

[1] Petta I Peene I Elewaut D Vereecke L De Bosscher K . Risks and benefits of corticosteroids in arthritic diseases in the clinic. Biochem Pharmacol. (2019) 165:112–25. doi: 10.1016/j.bcp.2019.04.009 30978323

[2] Clayton SA Jones SW Kurowska-Stolarska M Clark AR . The role of microRNAs in glucocorticoid action. J Biol Chem. (2018) 293:1865–74. doi: 10.1074/jbc.r117.000366 29301941 PMC5808749

[3] Reichardt SD Amouret A Muzzi C Vettorazzi S Tuckermann JP Lühder F . The role of glucocorticoids in inflammatory diseases. Cells. (2021) 10:2921. doi: 10.3390/cells10112921 34831143 PMC8616489

[4] Cain DW Cidlowski JA . Immune regulation by glucocorticoids. Nat Rev Immunol. (2017) 17:233–47. doi: 10.1038/nri.2017.1 28192415 PMC9761406

[5] Martinez GJ Appleton M Kipp ZA Loria AS Min B Hinds TD Jr. Glucocorticoids, their uses, sexual dimorphisms, and diseases: new concepts, mechanisms, and discoveries. Physiol Rev. (2024) 104:473–532. doi: 10.1152/physrev.00021.2023 37732829 PMC11281820

[6] Frank F Ortlund EA Liu X . Structural insights into glucocorticoid receptor function. Biochem Soc Trans. (2021) 49:2333–43. doi: 10.1042/bst20210419 34709368 PMC9274455

[7] Singh P Greulich F . Glucocorticoid receptor biology through the omics lens: context matters. Biol Chem. (2026). doi: 10.1515/hsz-2026-0147 42584960

[8] Wang C Zhong Y Tang M He X Xu S Zhang J . Navigating glucocorticoid resistance: From GR isoforms to nuclear condensates. Cell Invest. (2026) 2:100059. doi: 10.1016/j.clnves.2025.10005942574925

[9] Oakley RH Cidlowski JA . The biology of the glucocorticoid receptor: new signaling mechanisms in health and disease. J Allergy Clin Immunol. (2013) 132:1033–44. doi: 10.1016/j.jaci.2013.09.007 24084075 PMC4084612

[10] He Y Xu Y Zhang C Gao X Dykema KJ Martin KR . Identification of a lysosomal pathway that modulates glucocorticoid signaling and the inflammatory response. Sci Signal. (2011) 4:ra44. doi: 10.1126/scisignal.2001450 21730326 PMC3684214

[11] Yao W Dai W Jiang JX Lane NE . Glucocorticoids and osteocyte autophagy. Bone. (2013) 54:279–84. doi: 10.1016/j.bone.2013.01.034 23356984 PMC3784314

[12] Baschant U Tuckermann J . The role of the glucocorticoid receptor in inflammation and immunity. J Steroid Biochem Mol Biol. (2010) 120:69–75. doi: 10.1016/j.jsbmb.2010.03.058 20346397

[13] Kenney DL Benarroch EE . The autophagy-lysosomal pathway: General concepts and clinical implications. Neurology. (2015) 85:634–45. doi: 10.1212/WNL.0000000000001860 26203091

[14] Cruz-Topete D Cidlowski JA . One hormone, two actions: anti- and pro-inflammatory effects of glucocorticoids. Neuroimmunomodulation. (2015) 22:20–32. doi: 10.1159/000362724 PMC4243162 25227506

[15] Zhao Y Klionsky DJ Wang X Huang Q Deng Z Xiang J . The estrogen-autophagy axis: Insights into cytoprotection and therapeutic potential in cancer and infection. Int J Mol Sci. (2024) 25:12576. doi: 10.3390/ijms252312576 39684286 PMC11641569

[16] Nikoletopoulou V Markaki M Palikaras K Tavernarakis N . Crosstalk between apoptosis, necrosis and autophagy. Biochim Biophys Acta. (2013) 1833:3448–59. doi: 10.1016/j.bbamcr.2013.06.001 23770045

[17] Shimizu T Isik S Kamath N Yue Z . The emerging role and therapeutic targeting of autophagy-lysosome pathway in the pathogenesis of Parkinson's disease. Transl Neurodegener. (2026) 15:21. doi: 10.1186/s40035-026-00555-3 42093006 PMC13151188

[18] Lőrincz P Juhász G . Autophagosome-lysosome fusion. J Mol Biol. (2020) 432:2462–82. doi: 10.1016/j.jmb.2019.10.028 31682838

[19] Mizushima N Komatsu M . Autophagy: renovation of cells and tissues. Cell. (2011) 147:728–41. doi: 10.1016/j.cell.2011.10.026 22078875

[20] Clarke AJ Simon AK . Autophagy in the renewal, differentiation and homeostasis of immune cells. Nat Rev Immunol. (2019) 19:170–83. doi: 10.1038/s41577-018-0095-2 30531943

[21] Shang H Xu R Niu H Chai H Tang X Gou J . 20(S)-Protopanaxadiol regulating glucocorticoid receptor attenuates ischemic stroke injury by alleviating the autophagy-lysosomal pathway dysfunction and neuroinflammation. Phytomedicine. (2026) 150:157768. doi: 10.1016/j.phymed.2026.157768 41499936

[22] Keller CW Adamopoulos IE Lünemann JD . Autophagy pathways in autoimmune diseases. J Autoimmun. (2023) 136:103030. doi: 10.1016/j.jaut.2023.103030 37001435 PMC10709713

[23] Wang L Klionsky DJ Shen HM . The emerging mechanisms and functions of microautophagy. Nat Rev Mol Cell Biol. (2023) 24:186–203. doi: 10.1038/s41580-022-00529-z 36097284

[24] Tasset I Cuervo AM . Role of chaperone-mediated autophagy in metabolism. FEBS J. (2016) 283:2403–13. doi: 10.1111/febs.13677 26854402 PMC4935551

[25] Glick D Barth S Macleod KF . Autophagy: cellular and molecular mechanisms. J Pathol. (2010) 221:3–12. doi: 10.1002/path.2697 20225336 PMC2990190

[26] Li X He S Ma B . Autophagy and autophagy-related proteins in cancer. Mol Cancer. (2020) 19:12. doi: 10.1186/s12943-020-1138-4 31969156 PMC6975070

[27] Deploey N Van Moortel L Rogatsky I Peelman F De Bosscher K . The biologist's guide to the glucocorticoid receptor's structure. Cells. (2023) 12:1636. doi: 10.3390/cells12121636 37371105 PMC10297449

[28] Nicolaides NC . The human glucocorticoid receptor beta: From molecular mechanisms to clinical implications. Endocrinology. (2022) 163:bqac150. doi: 10.1210/endocr/bqac150 36059139

[29] He B Cruz-Topete D Oakley RH Xiao X Cidlowski JA . Human glucocorticoid receptor β regulates gluconeogenesis and inflammation in mouse liver. Mol Cell Biol. (2015) 36:714–30. doi: 10.1128/MCB.00908-15 PMC4760220 26711253

[30] Chen X He H . Expression profiles of glucocorticoid receptor α- and β-isoforms in diverse physiological and pathological conditions. Horm Metab Res. (2025) 57:359–65. doi: 10.1055/a-2619-5035 40404135

[31] Hasan MM Tory S . Association between glucocorticoid receptor beta and steroid resistance: A systematic review. Immun Inflammation Dis. (2024) 12:e1137. doi: 10.1002/iid3.1137 38270313 PMC10785191

[32] Hu Y Jiang Y Zhang Z Wang J Zhang B Gong L . Oncogenic activity of glucocorticoid receptor β is controlled by ubiquitination-dependent interaction with USP49 in glioblastoma cells. Mol Cancer Res. (2022) 20:92–101. doi: 10.1158/1541-7786.MCR-20-1068 PMC9398152 34610959

[33] Scheijen B . Molecular mechanisms contributing to glucocorticoid resistance in lymphoid malignancies. Cancer Drug Resist. (2019) 2:647–64. doi: 10.20517/cdr.2019.29 35582582 PMC8992511

[34] Wu T Shao Y Li X Wu T Yu L Liang J . NR3C1/Glucocorticoid receptor activation promotes pancreatic β-cell autophagy overload in response to glucolipotoxicity. Autophagy. (2023) 19:2538–57. doi: 10.1080/15548627.2023.2200625 37039556 PMC10392762

[35] Sato M Ueda E Konno A Hirai H Kurauchi Y Hisatsune A . Glucocorticoids negatively regulates chaperone mediated autophagy and microautophagy. Biochem Biophys Res Commun. (2020) 528:199–205. doi: 10.1016/j.bbrc.2020.04.132 32487317

[36] Weikum ER Knuesel MT Ortlund EA Yamamoto KR . Glucocorticoid receptor control of transcription: precision and plasticity via allostery. Nat Rev Mol Cell Biol. (2017) 18:159–74. doi: 10.1038/nrm.2016.152 28053348 PMC6257982

[37] Vettorazzi S Nalbantoglu D Gebhardt JCM Tuckermann J . A guide to changing paradigms of glucocorticoid receptor function-a model system for genome regulation and physiology. FEBS J. (2022) 289:5718–43. doi: 10.1111/febs.16100 34213830

[38] Bartlett AA Lapp HE Hunter RG . Epigenetic mechanisms of the glucocorticoid receptor. Trends Endocrinol Metab. (2019) 30:807–18. doi: 10.1016/j.tem.2019.07.003 31699238

[39] Weikum ER Okafor CD D'Agostino EH Colucci JK Ortlund EA . Structural analysis of the glucocorticoid receptor ligand-binding domain in complex with triamcinolone acetonide and a fragment of the atypical coregulator, small heterodimer partner. Mol Pharmacol. (2017) 92:12–21. doi: 10.1124/mol.117.108506 28396564 PMC5452056

[40] Vandevyver S Dejager L Libert C . On the trail of the glucocorticoid receptor: into the nucleus and back. Traffic. (2012) 13:364–74. doi: 10.1111/j.1600-0854.2011.01288.x 21951602

[41] Petta I Dejager L Ballegeer M Lievens S Tavernier J De Bosscher K . The interactome of the glucocorticoid receptor and its influence on the actions of glucocorticoids in combatting inflammatory and infectious diseases. Microbiol Mol Biol Rev. (2016) 80:495–522. doi: 10.1128/mmbr.00064-15 27169854 PMC4867367

[42] Vandevyver S Dejager L Tuckermann J Libert C . New insights into the anti-inflammatory mechanisms of glucocorticoids: an emerging role for glucocorticoid-receptor-mediated transactivation. Endocrinology. (2013) 154:993–1007. doi: 10.1210/en.2012-2045 23384835

[43] Scheschowitsch K Leite JA Assreuy J . New insights in glucocorticoid receptor signaling-more than just a ligand-binding receptor. Front Endocrinol (Lausanne). (2017) 8:16. doi: 10.3389/fendo.2017.00016 28220107 PMC5292432

[44] Meijsing SH . Mechanisms of glucocorticoid-regulated gene transcription. Adv Exp Med Biol. (2015) 872:59–81. doi: 10.1007/978-1-4939-2895-8_3 26215990

[45] McDowell IC Barrera A D'Ippolito AM Vockley CM Hong LK Leichter SM . Glucocorticoid receptor recruits to enhancers and drives activation by motif-directed binding. Genome Res. (2018) 28:1272–84. doi: 10.1101/gr.233346.117 30097539 PMC6120625

[46] de Theije CC Schols A Lamers WH Ceelen JJM van Gorp RH Hermans JJR . Glucocorticoid receptor signaling impairs protein turnover regulation in hypoxia-induced muscle atrophy in male mice. Endocrinology. (2018) 159:519–34. doi: 10.1210/en.2017-00603 29069356

[47] Wani WY Boyer-Guittaut M Dodson M Chatham J Darley-Usmar V Zhang J . Regulation of autophagy by protein post-translational modification. Lab Invest. (2015) 95:14–25. doi: 10.1038/labinvest.2014.131 25365205 PMC4454381

[48] Botti-Millet J Nascimbeni AC Dupont N Morel E Codogno P . Fine-tuning autophagy: from transcriptional to posttranslational regulation. Am J Physiol Cell Physiol. (2016) 311:C351–62. doi: 10.1152/ajpcell.00129.2016 27335173

[49] Liberman AC Antunica-Noguerol M Arzt E . Modulation of the glucocorticoid receptor activity by post-translational modifications. Nucl Receptor Res. (2014) 1:1–15. doi: 10.11131/2014/10108625391352

[50] Zeyen L Seternes OM Mikkola I . Crosstalk between p38 MAPK and GR signaling. Int J Mol Sci. (2022) 23:3322. doi: 10.3390/ijms23063322 35328742 PMC8953609

[51] Merkulov VM Klimova NV Merkulova TI . Glucocorticoid receptor: Translocation from the cytoplasm to the nuclei; chromatin and intranuclear chaperone cycles. Russian J Genet Appl Res. (2016) 6:297–306. doi: 10.1134/s2079059716030096

[52] Polman JA Welten JE Bosch DS de Jonge RT Balog J van der Maarel SM . A genome-wide signature of glucocorticoid receptor binding in neuronal PC12 cells. BMC Neurosci. (2012) 13:118. doi: 10.1186/1471-2202-13-118 23031785 PMC3519639

[53] Liu S Yao S Yang H Liu S Wang Y . Autophagy: Regulator of cell death. Cell Death Dis. (2023) 14:648. doi: 10.1038/s41419-023-06154-8 37794028 PMC10551038

[54] Klionsky DJ Petroni G Amaravadi RK Baehrecke EH Ballabio A Boya P . Autophagy in major human diseases. EMBO J. (2021) 40:e108863. doi: 10.15252/embj.2021108863 34459017 PMC8488577

[55] Relic B Charlier E Deroyer C Malaise O Neuville S Desoroux A . BAY 11-7085 induces glucocorticoid receptor activation and autophagy that collaborate with apoptosis to induce human synovial fibroblast cell death. Oncotarget. (2016) 7:23370–82. doi: 10.18632/oncotarget.8042 26993765 PMC5029633

[56] Yang Y Jia Y Sun Q Dong H Zhao R . White light emitting diode induces autophagy in hippocampal neuron cells through GSK-3-mediated GR and RORα pathways. Aging (Albany NY). (2019) 11:1832–49. doi: 10.18632/aging.101878 30923260 PMC6461168

[57] Cheng K Liu X Chen L Lv JM Qu FJ Pan XW . α-Viniferin activates autophagic apoptosis and cell death by reducing glucocorticoid receptor expression in castration-resistant prostate cancer cells. Med Oncol. (2018) 35:105. doi: 10.1007/s12032-018-1163-y 29904891

[58] Sun Q Yang Y Li X He B Jia Y Zhang N . Folate deprivation modulates the expression of autophagy- and circadian-related genes in HT-22 hippocampal neuron cells through GR-mediated pathway. Steroids. (2016) 112:12–9. doi: 10.1016/j.steroids.2016.04.010 27133904

[59] Qattan MY Bakker EY Rajendran R Chen DW Saha V Liu J . Differential regulation of cell death pathways by the microenvironment correlates with chemoresistance and survival in leukaemia. PloS One. (2017) 12:e0178606. doi: 10.1371/journal.pone.0178606 28582465 PMC5459454

[60] He B Zhao Y Xu L Gao L Su Y Lin N . The nuclear melatonin receptor RORα is a novel endogenous defender against myocardial ischemia/reperfusion injury. J Pineal Res. (2016) 60:313–26. doi: 10.1111/jpi.12312 26797926

[61] Xiong H Chen Z Lin B Xie B Liu X Chen C . Naringenin regulates FKBP4/NR3C1/NRF2 axis in autophagy and proliferation of breast cancer and differentiation and maturation of dendritic cell. Front Immunol. (2021) 12:745111. doi: 10.3389/fimmu.2021.745111 PMC8786807 35087512

[62] Wang X Weng Y Yao Y Shao S Mao L Wang J . Helicobacter pylori modulates macrophage polarization via the glucocorticoid receptor to promote gastric cancer cell proliferation. Tissue Cell. (2025) 96:103001. doi: 10.1016/j.tice.2025.103001 40472550

[63] Du L Liu W Aldana-Masangkay G Pozhitkov A Pichiorri F Chen Y . SUMOylation inhibition enhances dexamethasone sensitivity in multiple myeloma. J Exp Clin Cancer Res. (2022) 41:8. doi: 10.1186/s13046-021-02226-9 34983615 PMC8725350

[64] Kim J Kundu M Viollet B Guan KL . AMPK and mTOR regulate autophagy through direct phosphorylation of Ulk1. Nat Cell Biol. (2011) 13:132–41. doi: 10.1038/ncb2152 21258367 PMC3987946

[65] Holczer M Hajdú B Lőrincz T Szarka A Bánhegyi G Kapuy O . A double negative feedback loop between mTORC1 and AMPK kinases guarantees precise autophagy induction upon cellular stress. Int J Mol Sci. (2019) 20:5543. doi: 10.3390/ijms20225543 31703252 PMC6888297

[66] Nader N Ng SS Lambrou GI Pervanidou P Wang Y Chrousos GP . AMPK regulates metabolic actions of glucocorticoids by phosphorylating the glucocorticoid receptor through p38 MAPK. Mol Endocrinol. (2010) 24:1748–64. doi: 10.1210/me.2010-0192 20660302 PMC2940476

[67] Rogatsky I Logan SK Garabedian MJ . Antagonism of glucocorticoid receptor transcriptional activation by the c-Jun N-terminal kinase. Proc Natl Acad Sci USA. (1998) 95:2050–5. doi: 10.1073/pnas.95.5.2050 9482836 PMC19245

[68] Choi GE Lee HJ Chae CW Cho JH Jung YH Kim JS . BNIP3L/NIX-mediated mitophagy protects against glucocorticoid-induced synapse defects. Nat Commun. (2021) 12:487. doi: 10.1038/s41467-020-20679-y 33473105 PMC7817668

[69] Mylka V Deckers J Ratman D De Cauwer L Thommis J De Rycke R . The autophagy receptor SQSTM1/p62 mediates anti-inflammatory actions of the selective NR3C1/glucocorticoid receptor modulator compound A (CpdA) in macrophages. Autophagy. (2018) 14:2049–64. doi: 10.1080/15548627.2018.1495681 30215534 PMC6984772

[70] Boya P Reggiori F Codogno P . Emerging regulation and functions of autophagy. Nat Cell Biol. (2013) 15:713–20. doi: 10.1038/ncb2788 23817233 PMC7097732

[71] Ueno T Komatsu M . Monitoring autophagy flux and activity: Principles and applications. Bioessays. (2020) 42:e2000122. doi: 10.1002/bies.202000122 32851706

[72] Pugsley HR . Assessing autophagic flux by measuring LC3, p62, and LAMP1 co-localization using multispectral imaging flow cytometry. J Vis Exp. (2017):55637. doi: 10.3791/55637 28784946 PMC5612559

[73] Yang KC Sathiyaseelan P Ho C Gorski SM . Evolution of tools and methods for monitoring autophagic flux in mammalian cells. Biochem Soc Trans. (2018) 46:97–110. doi: 10.1042/bst20170102 29305410

[74] Klionsky DJ Abdel-Aziz AK Abdelfatah S Abdellatif M Abdoli A Abel S . Guidelines for the use and interpretation of assays for monitoring autophagy (4th edition)(1). Autophagy. (2021) 17:1–382. doi: 10.1080/15548627.2020.1797280 33634751 PMC7996087

[75] Alam MM Okazaki K Nguyen LTT Ota N Kitamura H Murakami S . Glucocorticoid receptor signaling represses the antioxidant response by inhibiting histone acetylation mediated by the transcriptional activator NRF2. J Biol Chem. (2017) 292:7519–30. doi: 10.1074/jbc.m116.773960 28314773 PMC5418050

[76] Lacher SE Krznarich J Levings DC Pathak SS Pufall M Yang YM . The glucocorticoid receptor inhibits NRF2-mediated expression of SLC7A11. Free Radic Biol Med. (2025) 241:53–63. doi: 10.1016/j.freeradbiomed.2025.09.008 40914322 PMC12897988

[77] Jain A Lamark T Sjøttem E Larsen KB Awuh JA Øvervatn A . p62/SQSTM1 is a target gene for transcription factor NRF2 and creates a positive feedback loop by inducing antioxidant response element-driven gene transcription. J Biol Chem. (2010) 285:22576–91. doi: 10.1074/jbc.m110.118976 20452972 PMC2903417

[78] Portuguez AS Grbesa I Tal M Deitch R Raz D Weismann R . Gene activation and repression by the glucocorticoid receptor are mediated by sequestering Ep300 and two modes of chromatin binding. Cold Spring Harbor Lab. (2019) bioRxiv. doi: 10.1101/764480

[79] Rao NA McCalman MT Moulos P Francoijs KJ Chatziioannou A Kolisis FN . Coactivation of GR and NFKB alters the repertoire of their binding sites and target genes. Genome Res. (2011) 21:1404–16. doi: 10.1101/gr.118042.110 21750107 PMC3166826

[80] Hiltunen J Aaltonen N Sohlberg H Kemppi L Paakinaho V . Molecular switch mediates the glucocorticoid receptor transition from tumor suppressor to oncogene in the prostate. bioRxiv. Cold Spring Harbor, NY: Cold Spring Harbor Laboratory. (2026). doi: 10.1101/2025.08.20.670078

[81] Jiang L Xu L Xie J Li S Guan Y Zhang Y . Inhibition of autophagy overcomes glucocorticoid resistance in lymphoid malignant cells. Cancer Biol Ther. (2015) 16:466–76. doi: 10.1080/15384047.2015.1016658 25778879 PMC4622576

[82] Sevilla LM Jiménez-Panizo A Alegre-Martí A Estébanez-Perpiñá E Caelles C Pérez P . Glucocorticoid resistance: Interference between the glucocorticoid receptor and the MAPK signalling pathways. Int J Mol Sci. (2021) 22:10049. doi: 10.3390/ijms221810049 34576214 PMC8465023

[83] Deng X Li Y Chen Y Hu Q Zhang W Chen L . Paeoniflorin protects hepatocytes from APAP-induced damage through launching autophagy via the MAPK/mTOR signaling pathway. Cell Mol Biol Lett. (2024) 29:119. doi: 10.1186/s11658-024-00631-4 39244559 PMC11380789

[84] Livingston MJ Zhang M Kwon SH Chen JK Li H Manicassamy S . Autophagy activates EGR1 via MAPK/ERK to induce FGF2 in renal tubular cells for fibroblast activation and fibrosis during maladaptive kidney repair. Autophagy. (2024) 20:1032–53. doi: 10.1080/15548627.2023.2281156 PMC11135847 37978868

[85] Peng J Lu N Hua S Song L Liu H . Unveiling the intricacies of autophagy in asthma: Unraveling novel therapeutic avenues. Front Biosci (Landmark Ed). (2024) 29:22. doi: 10.31083/j.fbl2901022 38287830

[86] Enweasor C Flayer CH Haczku A . Ozone-induced oxidative stress, neutrophilic airway inflammation, and glucocorticoid resistance in asthma. Front Immunol. (2021) 12:631092. doi: 10.3389/fimmu.2021.631092 33717165 PMC7952990

[87] Wang W Li JJ Foster PS Hansbro PM Yang M . Potential therapeutic targets for steroid-resistant asthma. Curr Drug Targets. (2010) 11:957–70. doi: 10.2174/138945010791591412 20412045

[88] Wu CJ Livak F Ashwell JD . The histone methyltransferase KMT2D maintains cellular glucocorticoid responsiveness by shielding the glucocorticoid receptor from degradation. J Biol Chem. (2024) 300:15. doi: 10.1016/j.jbc.2024.107581 39025450 PMC11350265

[89] Shibayama Y Alkhoury C Nemazanyy I N FH Cagnard N Girard M . Class 3 phosphoinositide 3-kinase promotes hepatic glucocorticoid receptor stability and transcriptional activity. Acta Physiol (Oxf). (2022) 235:e13793. doi: 10.1111/apha.13793 35094500 PMC9539506

[90] Blind RD Garabedian MJ . Differential recruitment of glucocorticoid receptor phospho-isoforms to glucocorticoid-induced genes. J Steroid Biochem Mol Biol. (2008) 109:150–7. doi: 10.1016/j.jsbmb.2008.01.002 18304804 PMC2699583

[91] Chen W Dang T Blind RD Wang Z Cavasotto CN Hittelman AB . Glucocorticoid receptor phosphorylation differentially affects target gene expression. Mol Endocrinol. (2008) 22:1754–66. doi: 10.1210/me.2007-0219 18483179 PMC2725771

[92] Lu NZ Cidlowski JA . Translational regulatory mechanisms generate N-terminal glucocorticoid receptor isoforms with unique transcriptional target genes. Mol Cell. (2005) 18:331–42. doi: 10.1016/j.molcel.2005.03.025 15866175

[93] Taniguchi Y Iwasaki Y Tsugita M Nishiyama M Taguchi T Okazaki M . Glucocorticoid receptor-beta and receptor-gamma exert dominant negative effect on gene repression but not on gene induction. Endocrinology. (2010) 151:3204–13. doi: 10.1210/en.2009-1254 20484466

[94] Rollins DA Kharlyngdoh JB Coppo M Tharmalingam B Mimouna S Guo Z . Glucocorticoid-induced phosphorylation by CDK9 modulates the coactivator functions of transcriptional cofactor GRIP1 in macrophages. Nat Commun. (2017) 8:1739. doi: 10.1038/s41467-017-01569-2 29170386 PMC5700924

[95] Guan WX Guo Y Chen ST Liu ZM Sun YY Zhao MW . Glucocorticoid-induced RPE injury in ARPE-19 cells: Association with excessive autophagy and AMPK/mTOR signaling. Int J Ophthalmol. (2026) 19:1657–66. doi: 10.18240/ijo.2026.09.01 42639231 PMC13501045

[96] Liberman AC Budziñski ML Sokn C Gobbini RP Steininger A Arzt E . Regulatory and mechanistic actions of glucocorticoids on T and inflammatory cells. Front Endocrinol (Lausanne). (2018) 9:235. doi: 10.3389/fendo.2018.00235 29867767 PMC5964134

[97] Ghislat G Lawrence T . Autophagy in dendritic cells. Cell Mol Immunol. (2018) 15:944–52. doi: 10.1038/cmi.2018.2 29578531 PMC6207777

[98] Harr MW McColl KS Zhong F Molitoris JK Distelhorst CW . Glucocorticoids downregulate Fyn and inhibit IP(3)-mediated calcium signaling to promote autophagy in T lymphocytes. Autophagy. (2010) 6:912–21. doi: 10.4161/auto.6.7.13290 20814235 PMC3039738

[99] Swerdlow S McColl K Rong Y Lam M Gupta A Distelhorst CW . Apoptosis inhibition by Bcl-2 gives way to autophagy in glucocorticoid-treated lymphocytes. Autophagy. (2008) 4:612–20. doi: 10.4161/auto.5920 18362516 PMC3625121

[100] Auger JP Zimmermann M Faas M Stifel U Chambers D Krishnacoumar B . Metabolic rewiring promotes anti-inflammatory effects of glucocorticoids. Nature. (2024) 629:184–92. doi: 10.1038/s41586-024-07282-7 38600378

[101] Ivy JR Carter RN Zhao JF Buckley C Urquijo H Rog-Zielinska EA . Glucocorticoids regulate mitochondrial fatty acid oxidation in fetal cardiomyocytes. J Physiol. (2021) 599:4901–24. doi: 10.1113/jp281860 34505639

[102] Viry E Paggetti J Baginska J Mgrditchian T Berchem G Moussay E . Autophagy: an adaptive metabolic response to stress shaping the antitumor immunity. Biochem Pharmacol. (2014) 92:31–42. doi: 10.1016/j.bcp.2014.07.006 25044308

[103] White E Lattime EC Guo JY . Autophagy regulates stress responses, metabolism, and anticancer immunity. Trends Cancer. (2021) 7:778–89. doi: 10.1016/j.trecan.2021.05.003 34112622 PMC8295230

[104] Garg AD Dudek AM Agostinis P . Autophagy-dependent suppression of cancer immunogenicity and effector mechanisms of innate and adaptive immunity. Oncoimmunology. (2013) 2:e26260. doi: 10.4161/onci.26260 24353910 PMC3862689

[105] Liu B Wen X Cheng Y . Survival or death: disequilibrating the oncogenic and tumor suppressive autophagy in cancer. Cell Death Dis. (2013) 4:e892. doi: 10.1038/cddis.2013.422 24176850 PMC3920945

[106] White E DiPaola RS . The double-edged sword of autophagy modulation in cancer. Clin Cancer Res. (2009) 15:5308–16. doi: 10.1158/1078-0432.ccr-07-5023 19706824 PMC2737083

[107] Xu H Zhao S Shi T Tian T Qin X He C . Immunogenic cell death-primed autophagosome vaccines drive dendritic cell cross-presentation and suppress colon cancer metastasis. J Control Release. (2026) 393:114827. doi: 10.1016/j.jconrel.2026.114827 41856327

[108] Carleton G Lum JJ . Autophagy metabolically suppresses CD8+ T cell antitumor immunity. Autophagy. (2019) 15:1–2. doi: 10.1080/15548627.2019.1628545 31170865 PMC6693469

[109] Xie C Zhou X Liang C Li X Ge M Chen Y . Apatinib triggers autophagic and apoptotic cell death via VEGFR2/STAT3/PD-L1 and ROS/Nrf2/p62 signaling in lung cancer. J Exp Clin Cancer Res. (2021) 40:266. doi: 10.1186/s13046-021-02069-4 34429133 PMC8385858

[110] Xin Z Xu R Dong Y Jin S Ge X Shen X . Impaired autophagy-mediated macrophage polarization contributes to age-related hyposalivation. Cell Prolif. (2024) 57:e13714. doi: 10.1111/cpr.13714 39004782 PMC11628751

[111] Xiao S Li G Tan M Liu W Li W . Loss of BACH1 improves osteogenic differentiation in glucocorticoid-induced hBMSCs through restoring autophagy. BMC Musculoskelet Disord. (2024) 25:665. doi: 10.1186/s12891-024-07761-y 39182017 PMC11344390

[112] Wang J Wang R Yang J Yang X Hu S Wang H . Glucocorticoids differentially regulate the innate immune responses of TLR4 and the cytosolic DNA sensing pathway. Int Immunopharmacol. (2017) 47:190–8. doi: 10.1016/j.intimp.2017.03.022 28415024

[113] Xu J Wang B Ao H . Corticosterone effects induced by stress and immunity and inflammation: mechanisms of communication. Front Endocrinol (Lausanne). (2025) 16:1448750. doi: 10.3389/fendo.2025.1448750 40182637 PMC11965140

[114] Bongiovanni B Mata-Espinosa D D'Attilio L Leon-Contreras JC Marquez-Velasco R Bottasso O . Effect of cortisol and/or DHEA on THP1-derived macrophages infected with Mycobacterium tuberculosis. Tuberculosis (Edinb). (2015) 95:562–9. doi: 10.1016/j.tube.2015.05.011 26099547

[115] Yu XH Xu XM Zhang SX . Low-dose dexamethasone promotes osteoblast viability by activating autophagy via the SGK1/FOXO3a signaling pathway. Cell Biol Int. (2023) 47:669–78. doi: 10.1002/cbin.11971 PMC10108317 36453461

[116] Han Y Zhang L Xing Y Zhang L Chen X Tang P . Autophagy relieves the function inhibition and apoptosis-promoting effects on osteoblast induced by glucocorticoid. Int J Mol Med. (2018) 41:800–8. doi: 10.3892/ijmm.2017.3270 PMC5752167 29207032

[117] Juszczak GR Stankiewicz AM . Glucocorticoids, genes and brain function. Prog Neuro-Psychopharmacol Biol Psychiatry. (2018) 82:136–68. doi: 10.1016/j.pnpbp.2017.11.020 29180230

[118] Flammer JR Rogatsky I . Minireview: Glucocorticoids in autoimmunity: unexpected targets and mechanisms. Mol Endocrinol. (2011) 25:1075–86. doi: 10.1210/me.2011-0068 21511881 PMC5417249

[119] Jang YJ Kim JH Byun S . Modulation of autophagy for controlling immunity. Cells. (2019) 8:138. doi: 10.3390/cells8020138 30744138 PMC6406335

[120] Debnath J Gammoh N Ryan KM . Autophagy and autophagy-related pathways in cancer. Nat Rev Mol Cell Biol. (2023) 24:560–75. doi: 10.1038/s41580-023-00585-z 36864290 PMC9980873

[121] Babaei G Aziz SG Jaghi NZZ . EMT, cancer stem cells and autophagy; The three main axes of metastasis. BioMed Pharmacother. (2021) 133:110909. doi: 10.1016/j.biopha.2020.110909 33227701

[122] Liu L Aleksandrowicz E Schönsiegel F Gröner D Bauer N Nwaeburu CC . Dexamethasone mediates pancreatic cancer progression by glucocorticoid receptor, TGFβ and JNK/AP-1. Cell Death Dis. (2017) 8:e3064. doi: 10.1038/cddis.2017.455 28981109 PMC5680577

[123] Liu L Han S Xiao X An X Gladkich J Hinz U . Glucocorticoid-induced microRNA-378 signaling mediates the progression of pancreatic cancer by enhancing autophagy. Cell Death Dis. (2022) 13:1052. doi: 10.1038/s41419-022-05503-3 36535942 PMC9763328

[124] Galluzzi L Pietrocola F Bravo-San Pedro JM Amaravadi RK Baehrecke EH Cecconi F . Autophagy in malignant transformation and cancer progression. EMBO J. (2015) 34:856–80. doi: 10.15252/embj.201490784 25712477 PMC4388596

[125] Pérez-Hernández M Arias A Martínez-García D Pérez-Tomás R Quesada R Soto-Cerrato V . Targeting autophagy for cancer treatment and tumor chemosensitization. Cancers (Basel). (2019) 11:1599. doi: 10.3390/cancers11101599 PMC6826429 31635099

[126] Guo Z Liu JL Fang YN Guo XM Ma CL Wang JW . Solasodine binds to glucocorticoid receptor to ameliorate airway remodeling and excessive autophagy in bronchial smooth muscle cells for allergic asthma. Toxicol Appl Pharmacol. (2025) 498:117313. doi: 10.1016/j.taap.2025.117313 40154577

[127] Gewirtz DA . The challenge of developing autophagy inhibition as a therapeutic strategy. Cancer Res. (2016) 76:5610–4. doi: 10.1158/0008-5472.can-16-0722 27634767 PMC5050139

[128] De Bosscher K . Selective glucocorticoid receptor modulators. J Steroid Biochem Mol Biol. (2010) 120:96–104. doi: 10.1016/j.jsbmb.2010.02.027 20206690

[129] Wnuk A Kajta M . Steroid and xenobiotic receptor signalling in apoptosis and autophagy of the nervous system. Int J Mol Sci. (2017) 18:2394. doi: 10.3390/ijms18112394 29137141 PMC5713362

[130] Shi H Li B Zhang D Han H He H Zhu J . Autophagy inhibition mediated by intrauterine miR-1912-3p/CTSD programming participated in the susceptibility to osteoarthritis induced by prenatal dexamethasone exposure in male adult offspring rats. FASEB J. (2023) 37:e23011. doi: 10.1096/fj.202300022rr 37249374

[131] Zhou H Mehta S Srivastava SP Grabinska K Zhang X Wong C . Endothelial cell-glucocorticoid receptor interactions and regulation of Wnt signaling. JCI Insight. (2020) 5:e131384. doi: 10.1172/jci.insight.131384 32051336 PMC7098785

[132] Liu B Zhou H Zhang T Gao X Tao B Xing H . Loss of endothelial glucocorticoid receptor promotes angiogenesis via upregulation of Wnt/β-catenin pathway. Angiogenesis. (2021) 24:631–45. doi: 10.1007/s10456-021-09773-x 33650028 PMC8292305

[133] Han H Shi H Jiang L Zhang D Wang H Li J . Autophagy activated by GR/miR-421-3p/mTOR pathway as a compensatory mechanism participates in chondrodysplasia induced by prenatal caffeine exposure in male fetal rats. Toxicol Lett. (2024) 397:141–50. doi: 10.1016/j.toxlet.2024.05.010 38759937

[134] Anderson KC Kyle RA Rajkumar SV Stewart AK Weber D Richardson P . Clinically relevant end points and new drug approvals for myeloma. Leukemia. (2008) 22:231–9. doi: 10.1038/sj.leu.2405016 17972944

[135] Li X Wu T Chen W Zhang J Jiang Y Deng J . Andrographolide acts with dexamethasone to inhibit the growth of acute lymphoblastic leukemia CEM-C1 cells via the regulation of the autophagy-dependent PI3K/AKT/mTOR signaling pathway. BioMed Rep. (2024) 20:43. doi: 10.3892/br.2024.1731 38357243 PMC10865295

[136] Ozpolat B Benbrook DM . Targeting autophagy in cancer management - strategies and developments. Cancer Manag Res. (2015) 7:291–9. doi: 10.2147/cmar.s34859 26392787 PMC4573074

[137] Pierro J Hogan LE Bhatla T Carroll WL . New targeted therapies for relapsed pediatric acute lymphoblastic leukemia. Expert Rev Anticancer Ther. (2017) 17:725–36. doi: 10.1080/14737140.2017.1347507 28649891 PMC6028000

[138] Bedewy AM El-Maghraby SM Kandil NS El-Bendary WR . The prognostic value of glucocorticoid receptors for adult acute lymphoblastic leukemia. Blood Res. (2015) 50:235–41. doi: 10.5045/br.2015.50.4.235 26770951 PMC4705049

[139] Xu JY Luo JM . Association between BIM gene and glucocorticoid resistance in children with acute lymphoblastic leukemia. Zhongguo Dang Dai Er Ke Za Zhi. (2017) 19:945–9. doi: 10.7499/j.issn.1008-8830.2017.08.018 PMC7390050 28774373

[140] Wandler AM Huang BJ Craig JW Hayes K Yan H Meyer LK . Loss of glucocorticoid receptor expression mediates *in vivo* dexamethasone resistance in T-cell acute lymphoblastic leukemia. Leukemia. (2020) 34:2025–37. doi: 10.1038/s41375-020-0748-6 32066867 PMC7440098

[141] Yuan N Song L Zhang S Lin W Cao Y Xu F . Bafilomycin A1 targets both autophagy and apoptosis pathways in pediatric B-cell acute lymphoblastic leukemia. Haematologica. (2015) 100:345–56. doi: 10.3324/haematol.2014.113324 PMC4349273 25512644

[142] Galluzzi L Bravo-San Pedro JM Levine B Green DR Kroemer G . Pharmacological modulation of autophagy: therapeutic potential and persisting obstacles. Nat Rev Drug Discov. (2017) 16:487–511. doi: 10.1038/nrd.2017.22 28529316 PMC5713640

[143] Loos B Klionsky DJ Du Toit A Hofmeyr JS . On the relevance of precision autophagy flux control *in vivo* - Points of departure for clinical translation. Autophagy. (2020) 16:750–62. doi: 10.1080/15548627.2019.1687211 PMC7138200 31679454

[144] Sundahl N Bridelance J Libert C De Bosscher K Beck IM . Selective glucocorticoid receptor modulation: New directions with non-steroidal scaffolds. Pharmacol Ther. (2015) 152:28–41. doi: 10.1016/j.pharmthera.2015.05.001 25958032

[145] Xia X Kar R Gluhak-Heinrich J Yao W Lane NE Bonewald LF . Glucocorticoid-induced autophagy in osteocytes. J Bone Miner Res. (2010) 25:2479–88. doi: 10.1002/jbmr.160 20564240 PMC3179284

[146] Jia J Yao W Guan M Dai W Shahnazari M Kar R . Glucocorticoid dose determines osteocyte cell fate. FASEB J. (2011) 25:3366–76. doi: 10.1096/fj.11-182519 21705669 PMC3177583

[147] Molitoris JK McColl KS Swerdlow S Matsuyama M Lam M Finkel TH . Glucocorticoid elevation of dexamethasone-induced gene 2 (Dig2/RTP801/REDD1) protein mediates autophagy in lymphocytes. J Biol Chem. (2011) 286:30181–9. doi: 10.1074/jbc.m111.245423 21733849 PMC3191057

[148] Hu W Jiang C Kim M Yang W Zhu K Guan D . Individual-specific functional epigenomics reveals genetic determinants of adverse metabolic effects of glucocorticoids. Cell Metab. (2021) 33:1592–1609.e7. doi: 10.1016/j.cmet.2021.06.004 34233159 PMC8340270

[149] Colina M Campana G . The aspiration of precision glucocorticoid pharmacotherapy in immune-mediated diseases: where do we stand in integrating pharmacogenomics and biomarkers for individualized treatment? Front Immunol. (2026) 17:1889058. doi: 10.3389/fimmu.2026.1889058 42707746 PMC13548042

[150] Bonapace L Bornhauser BC Schmitz M Cario G Ziegler U Niggli FK . Induction of autophagy-dependent necroptosis is required for childhood acute lymphoblastic leukemia cells to overcome glucocorticoid resistance. J Clin Invest. (2010) 120:1310–23. doi: 10.1172/jci39987 20200450 PMC2846044

